# Site-Directed Mutagenesis of the Carotenoid Isomerase Gene *BnaCRTISO* Alters the Color of Petals and Leaves in *Brassica napus* L.

**DOI:** 10.3389/fpls.2022.801456

**Published:** 2022-02-10

**Authors:** Huailin Li, Kaidi Yu, Olalekan Amoo, Yalun Yu, Mixia Guo, Songyue Deng, Mengting Li, Limin Hu, Jingzhen Wang, Chuchuan Fan, Yongming Zhou

**Affiliations:** ^1^National Key Laboratory of Crop Genetic Improvement, Huazhong Agricultural University, Wuhan, China; ^2^Hubei Hongshan Laboratory, Wuhan, China

**Keywords:** *Brassica napus*, flower color, *BnaCRTISO*, gene editing, carotenoid

## Abstract

The diversity of petal and leaf color can improve the ornamental value of rapeseed and promote the development of agriculture and tourism. The two copies of carotenoid isomerase gene (*BnaCRTISO*) in *Brassica napus* (*BnaA09.CRTISO* and *BnaC08.CRTISO*) was edited using the CRISPR/Cas9 system in the present study. The mutation phenotype of creamy white petals and yellowish leaves could be recovered only in targeted mutants of both *BnaCRTISO* functional copies, indicating that the redundant roles of *BnaA09.CRTISO* and *BnaC08.CRTISO* are vital for the regulation of petal and leaf color. The carotenoid content in the petals and leaves of the *BnaCRTISO* double mutant was significantly reduced. The chalcone content, a vital substance that makes up the yellow color, also decreased significantly in petals. Whereas, the contents of some carotenes (lycopene, α-carotene, γ-carotene) were increased significantly in petals. Further, transcriptome analysis showed that the targeted mutation of *BnaCRTISO* resulted in the significant down-regulation of important genes *BnaPSY* and *BnaC4H* in the carotenoid and flavonoid synthesis pathways, respectively; however, the expression of other genes related to carotenes and xanthophylls synthesis, such as *BnaPDS3*, *BnaZEP*, *BnaBCH1* and *BCH2*, was up-regulated. This indicates that the molecular mechanism regulating petal color variation in *B. napus* is more complicated than those reported in *Arabidopsis* and other *Brassica* species. These results provide insight into the molecular mechanisms underlying flower color variation in rapeseed and provides valuable resources for rapeseed breeding.

## Introduction

Flower color is considered one of the major attractants for pollen transmission in nature. Insects can recognize different flower colors through visual signals and transmit targeted pollen between flowers (Cazzonelli, 2011; Nisar et al., 2015). It is generally recognized that flower color is determined by three main pigments: carotenoids, flavonoids, and betalains (Grotewold, 2006). Carotenoids mainly provide orange and red color, flavonoids provide yellow, and betalains provide a few other colors (Cazzonelli, 2011). They are fat-soluble terpenoids synthesized *via* the isoprene pathway and have been found in various plants and animals (Cunningham and Gantt, 1998). Carotenoids are important pigments determining the color of fruits, vegetables, and flowers (Lv et al., 2015; Zhang et al., 2015; Li X. et al., 2018). They are found mainly in leaf, flower, fruit, and root tissues and play a vital role during plant development, such as protecting the plants against photo-oxidative damage (Holt et al., 2005; Sun et al., 2018). Carotenoids are also the precursors for the biosynthesis of vitamin A, the plant hormones abscisic acid (ABA) and strigolactone (Walter and Strack, 2011).

The biosynthetic pathway of carotenoid in higher plants has been elucidated, and many genes encoding key enzymes involved in this pathway have been successfully cloned. Based on the presence or absence of oxygen in their molecular structure, carotenoids can be divided into two categories, i.e., carotenes and xanthophylls. Carotenes contain carbon and hydrogen in their molecular structure, such as phytoene, lycopene, and α-carotene; xanthophylls contain oxygen, such as lutein, zeaxanthin, neoxanthin (Montserrat et al., 2015). In general, carotenoids in plants use GGPP (Geranylgeranyl pyrophosphate) as the synthetic substrate except for a few plants (Fraser and Bramley, 2004; Matsufuji et al., 2007). In *Arabidopsis thaliana*, the biosynthesis of carotenoid is regulated by ten enzymes and eleven genes (Patrick et al., 2018).

Carotenoid isomerase (CRTISO) converts the yellow colored prolycopene into the red colored all-*trans* lycopene in the carotenoid synthesis pathway (Breitenbach and Sandmann, 2005). Previous studies have shown that down-regulation of *CRTISO* gene expression in citrus can promote the accumulation of β-carotene (Kato et al., 2007). It was also reported that the expression of *CRTISO* gene could increase carotenoid accumulation in the endosperm of maize seeds (Wurtzel, 2009), implying that the expression of *CRTISO* gene has a certain correlation with the accumulation of carotenoids in plants. At present, the *CRTISO* gene has been cloned in tomato (Isaacson, 2002), maize (Wurtzel, 2009), melon (Galpaz et al., 2013), and *A. thaliana* (Hyoungshin, 2002). But there is no relevant research report on this gene in rapeseed.

Rapeseed is the third-largest oilseed crop worldwide with multi-function application, which are widely used as edible vegetable oil, vegetables, fodder, biofuel and nectar, improvement of saline and alkaline soils (Liu et al., 2020). The distinctive flower color of rapeseed also shows high ornamental value, which has received increased attention in China. The typical flower color of rapeseed is yellow, but there are also reports of some variant flower colors, such as white, yellowish and orange. Numerous genetic analysis of flower color have indicated that this trait is little affected by environmental factors and shows dominant or incompletely dominant inheritance (Quazi, 1988; Zhang et al., 2010; Huang et al., 2014). Up to date, several genes controlling flower color have been reported in *B. napus*. The white flower color is controlled by a single dominant gene, *BnaC3.CCD4*, which encodes a carotenoid cleavage dioxygenase and is involved in carotenoid degradation (Zhang et al., 2015; Han et al., 2019). The insertion of a CACTA-like transposable element in *BnaC3.CCD4* leads to its loss-of-function and a subsequently enhanced accumulation of carotenoids; thus, results in a petal color transition from white to yellow (Zhang et al., 2015; Han et al., 2019). Gene silencing of two genes, *BnaA09.ZEP* and *BnaC09.ZEP*, confers the change in flower color from yellow to orange (Liu et al., 2020). These two genes are homologous to the nuclear-encoded plastid enzyme zeaxanthin epoxidase (AtZEP) and participate in carotenoid biosynthesis. Recently, Zhao et al. (2021) reported that a yellowish-white flower trait is controlled by a single recessive gene, *BnaA08.PDS3*, which encodes a phytoene desaturase 3 (Zhao et al., 2021). A C-to-T substitution in the coding region of *BnaA08.PDS3* results in a premature translation termination and a subsequent decreased carotenoid biosynthesis; thus, changing the flower color from yellow to yellowish-white. Other researchers used cell fusion technology to obtain the fusion plant with white flower trait (47 chromosomes). When the chromosomes were reduced to 38 by backcrossing, the petal color changed from white to yellow (Sakai, 1995). Therefore, until now, the genes and molecular mechanisms regulating flower color in rapeseed have not been fully elucidated.

In recent years, sequence-specific nucleases (SSNs) have been demonstrated to be an amazing tool for improving crops *via* site-specific genome editing, and CRISPR/Cas9 is considered the most simple and efficient SSN. The CRISPR/Cas9 system has been effectively utilized in rapeseed to produce the targeted mutations for the improvement of numerous agronomic traits (Braatz et al., 2017; Yang et al., 2017, 2018; Hu et al., 2018; Li C. et al., 2018; Zhai et al., 2019; Ahmar et al., 2021).

Hence, we utilized the CRISPR/Cas9 system to generate efficient knockouts of *CRTISO* homeologs with stable transformation in rapeseed. In the T_1_, T_2,_ and T_3_ generations, mutants containing the desired gene modification were obtained by segregation. The transcriptomic analysis and metabolite profiling of *BnaCRTISO* mutant plants were used in this current study to investigate the molecular mechanisms that regulate the petal color in *B. napus*. This study provided valuable germplasm resources for the innovation of different petal color varieties in rapeseed and offered a new way to improve polypoid crops.

## Materials and Methods

### Plant Materials

In this study, *B. napus* pure line J9707 was used as the donor plants for transformation, and the seeds were obtained from the National Engineering Research Center of Rapeseed, Wuhan, China. The flowers on the primary inflorescence were marked at anthesis, and the petals with different colors were collected for transcriptomic analysis and metabolite profiling.

### Construction of the CRISPR/Cas9 Vector and Plant Transformation

**FIGURE 1:**
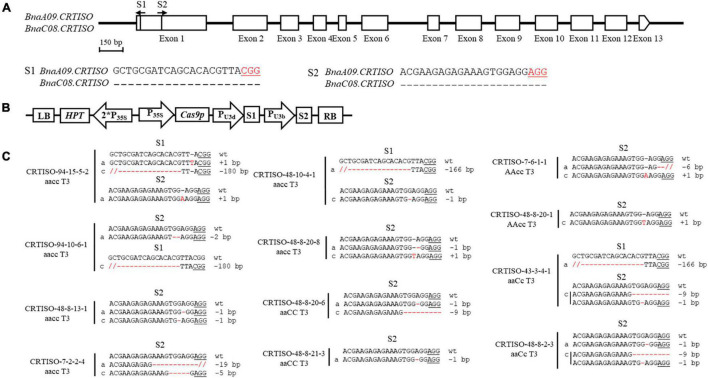
CRISPR/Cas9-induced null mutants of *BnaCRTISO* in *B. napus*. **(A)** The *BnaCRTISO* gene model includes thirteen exons (box) separated by twelve introns (represented by the solid line). The vertical line in the gene model indicates the target site, and the arrow indicates the sgRNA direction. The target sequences are shown with the protospacer adjacent motif (PAM) underlined. **(B)** The CRISPR/Cas9 construct houses the following: a hygromycin resistance cassette consisting of the hygromycin phosphotransferase coding sequence driven by the cauliflower mosaic virus 35S promoter; a Cas9 expression cassette comprising the sequence encoding Cas9 driven by 35S promoter; and two sgRNAs (S1-S2) driven by the U3d and U3b promoters from *Arabidopsis*. **(C)** Sequences at the sgRNA target sites of *BnaCRTISO* homozygous mutants in the T_3_ generation. The PAM is underlined, and nucleotide indels are marked in red, with details labeled at right, “a” and “c” represent the mutated alleles of the target gene on *BnaA09.CRTISO* and *BnaC08.CRTISO*, respectively. “*aaCC*,” “*AAcc*,” and “*aacc*” represent homozygous mutations of the target gene in *BnaA09.CRTISO*, *BnaC08.CRTISO* and both copies, respectively.

The binary pYLCRIPSR/Cas9 multiplex genome targeting vector system was utilized for gene editing in this study (Ma et al., 2015). The selection of sequence-specific sgRNAs in the target gene, CRISPR/Cas9 construct assembly, and *Agrobacterium tumefaciens*-mediated hypocotyl transformation in *B. napus* were conducted as previously described (Hu et al., 2018). The oligos employed in constructing the sgRNA vectors are listed in Supplementary Table 1. The resulting construct is described in detail in Figure 1B.

### Identification of Transgenic Plants and Potential Off-Targets

The transgenic plants were screened by hygromycin selection (25 mg/L). Then, the presence of the T-DNA in the construct was assessed by PCR using the specific primer pairs PB-L/PB-R (Supplementary Table 1).

The targeted mutations were determined in transgenic plants using the high-throughput tracking of mutations (Hi-TOM) platform (Zhai et al., 2019). Target-specific and barcoding PCR (two rounds of PCR) were performed to amplify the genomic region encompassing the specific targets of each independent sample, and the resulting PCR products were mixed in equal amounts and purified for next-generation sequencing (the Illumina HiSeq platform at the Novogene Bioinformatics Institute, Beijing, China). The sequencing data was then decoded using a corresponding online tool to track the mutations of the target sites^1^. The potential off-target sites were identified using CRISPR-P2.0^2^. The primers used to detect targeted and potential off-target mutations are listed in Supplementary Table 1.

### RNA Extraction and Quantitative Real-Time PCR

Total RNA was prepared using the EasyPure Plant RNA Kit (TransGen Biotech, Beijing, China), and cDNA was synthesized using the Transcript RT Kit (TransGen Biotech). The qRT-PCR was carried out using the TransStart Top Green qPCR SuperMix Kit (TransGen Biotech) on a CFX384 Real-Time System (Bio-Rad). Relative quantification was performed using the comparative cycle threshold method. The relative amount of PCR product that was amplified using the designed primer sets (Supplementary Table 1) was normalized to the reference genes, *BnaACT2* and *BnaUBC9*.

### RNA-Seq Transcriptomic Analysis

Flower tissues were sampled with three biological replicates. Petals were gently hand dissected from the flower on dry ice, immediately frozen immediately in liquid nitrogen, and stored at −80°C until total RNA extraction.

RNA extraction, cDNA library construction, sequencing, quality control, and read mapping to the reference genome, identification of differentially expressed genes (DEGs), and GO and KEGG pathway enrichment analysis of DEGs were performed using previously established procedure (Shahid et al., 2019; Zhai et al., 2020). Fragments per kilobase of transcript per million mapped reads (FPKM) were calculated as a measure of the level of gene expression. Genes with a false discovery rate (FDR) ≤ 0.05 and an absolute value of log_2_ fold change ≥ 1 between mutant and wild type (WT) were defined as DEGs. The raw sequence data were deposited in the NCBI Sequence Read Archive (PRJNA749083).

### Metabolite Profiling

Metabolites were extracted from petals (500 mg dry weight) with three biological replicates and were analyzed using LC-ESI-MS/MS system at the Metware Biotechnology Co., Ltd. (Wuhan, China). Flavonoids were extracted using the same method at the National Engineering Research Center of Rapeseed (Huazhong Agricultural University, Wuhan, China). The sample extraction and metabolic analysis were explicitly done as previously described (Zhai et al., 2020).

### Measurement of Carotenoid and Chlorophyll

For carotenoids analysis, the petals were sampled from double mutants (CRTISO-94-15-5-2, CRTISO-48-8-13-1, CRTISO-7-2-2-4), heterozygous mutant (CRTISO-43-3-4-1, CRTISO-48-8-2-3, CRTISO-48-8-20-5) and WT. Carotenoid pigments extraction and analysis were performed using LC-MS/MS system, as previously described (Lee, 2001; Saladié et al., 2014). Carotenoids were identified based on retention times and absorption spectra as compared to standards. Peak areas were recorded at 286, 348, 473, and 450 nm for phytoene, phytofluene, lycopene, and others, respectively (Xu et al., 2006). The carotenoid levels were quantified using calibration curves prepared with appropriate standards. At least three independent extractions were conducted per sample.

For chlorophyll analysis, the leaves was extracted from double mutants (CRTISO-94-15-5-2, CRTISO-48-8-13-1, CRTISO-7-2-2-4) and WT and quantified using a spectrophotometer, as described previously (Becker, 1994). At least three independent extractions were conducted per sample.

### Subcellular Localization

The cDNA sequences of BnaA09.CRTISO and BnaC08.CRTISO without the termination codon were amplified from J9707 using primers CRTISO-15/16 and CRTISO-18/19, respectively (Supplementary Table 1). The amplified cDNA fragments were independently cloned into the pMDC83 vector between the *Pac*I and the *Asc*I site, to generate a C-terminal fusion with GFP under control of the cauliflower mosaic virus 35S promoter. The fused construct was introduced into *Nicotiana benthamiana* plants by transient *Agrobacterium* transformation. Samples were observed with a Leica TCSST2 confocal laser microscope (Nikon D760, Tokyo, Japan).

## Results

### Molecular Cloning and Characterization of *CRTISO* Homologs in *B. napus*

Previous studies revealed that the *CRTISO* gene is essential for regulating carotenoid content and is highly conserved in many plants (Galpaz et al., 2013; Su et al., 2015; Li et al., 2020; Sun et al., 2020). The carotenoid content of a flower directly determines the color of the petals (Su et al., 2015; Zhang et al., 2015; Liu et al., 2020; Zhao et al., 2021). Thus, *CRTISO* is one of the ideal candidates for generating different flower colors in rapeseed. There are two *CRTISO* copies (*BnaA09g49740D* and *BnaC08g44970D*, designated as *BnaA09.CRTISO* and *BnaC08.CRTISO*, respectively) in *B. napus*. In order to check for putative mutations in these target genes, we confirmed their genomic DNA and cDNA sequences in the *B. napus* pure line J9707. Compared to *Arabidopsis* AtCRTISO, the predicted amino acid sequences of both BnaCRTISO contain an important conserved domain, “the amine oxidase” (Supplementary Figures 1, 2). which meanings *BnaA09.CRTISO* and *BnaC08.CRTISO* can produce functional amine oxidase proteins.

*BnaA09.CRTISO* and *BnaC08.CRTISO* was 87.94% identical at the nucleotide level and shared 98.64% amino acid identity, suggesting that both genes encode enzymes with similar functions. The sequence alignment of both *BnaCRTISO* gene copies revealed that polymorphisms distinguished their origins (Supplementary Figure 3).

Phylogenetic analysis indicated that all *Brassica* genes were clustered together with *AtCRTISO* and *CRTISO* homologs in other species were clustered on another branch except in tomato (*SolCRTISO*), suggesting that the *CRTISO* gene is differentiated among different species (Supplementary Figure 4). *BnaA09.CRTISO* and *BnaC08.CRTISO* were closely related to their homologs in *B. rapa* and *B. oleracea*, respectively (Supplementary Figure 4), which is in line with their origination from two diploid progenitors.

### Expression Analysis of the *BnaCRTISO* Gene

Analysis of mRNA accumulation patterns of both *BnaCRTISO* copies based on the public RNA-seq data in yellow-flower rapeseed line showed their expression profiles (Supplementary Figure 5A). In all cases, *BnaA09.CRTISO* and *BnaC08.CRTISO* had the highest expression levels in the bud, followed by leaf, and the expression levels of both copies in various tissues were comparable. Thus, we further confirmed that the *B. napus* genome contains two functional *AtCRTISO* homologs, *BnaA09.CRTISO* and *BnaC08.CRTISO*.

The expression level of both *BnaCRTISO* copies in J9707 was further examined using qRT-PCR with RNA samples extracted from the petal (Supplementary Figure 5B). Consistent with the public RNA-seq data, *BnaC08.CRTISO* had a significantly higher expression level than *BnaA09.CRTISO* in the J9707 petal (Supplementary Figure 5A).

### Creation of CRISPR/Cas9-Targeted Mutations in *BnaCRTISO*

To generate CRISPR/Cas9-induced knockout mutations in the functional copies of *BnaCRTISO*, two sgRNAs were designed using the CRISPR-P program (Lei et al., 2014). The two sgRNAs [sgRNA1 (S1) and sgRNA2 (S2)] were designed to target the amine oxidase domain, which will induce mutations in the functional domain of the *BnaCRTISO* gene resulting in the formation of non-functional protein (Figure 1A and Supplementary Figure 3). The designed sgRNAs matched well with both *BnaA09.CRTISO* and *BnaC08.CRTISO* copies (Figure 1A). A CRISPR/Cas9 construct containing these two sgRNAs with Cas9 driven by the cauliflower mosaic virus 35S promoter (Figure 1B) was produced based on the CRISPR/Cas9 multiplex genome-editing vector previously described by Yang et al. (2017). The resulting construct was transformed into J9707 using *Agrobacterium*-mediated transformation. A total of 105 seedlings were regenerated, of which 96 were transgenic positive. And 72 targeted mutants were identified by Sanger DNA sequencing of the PCR products encompassing the target sites, with 37 plants showing a visible knockout phenotype (i.e., creamy white flower; Supplementary Table 2). The overall editing efficiency of the T_0_ generations is 75.00%, of which the editing efficiency of S1 and S2 were 16.39 and 73.22%, respectively.

**FIGURE 2:**
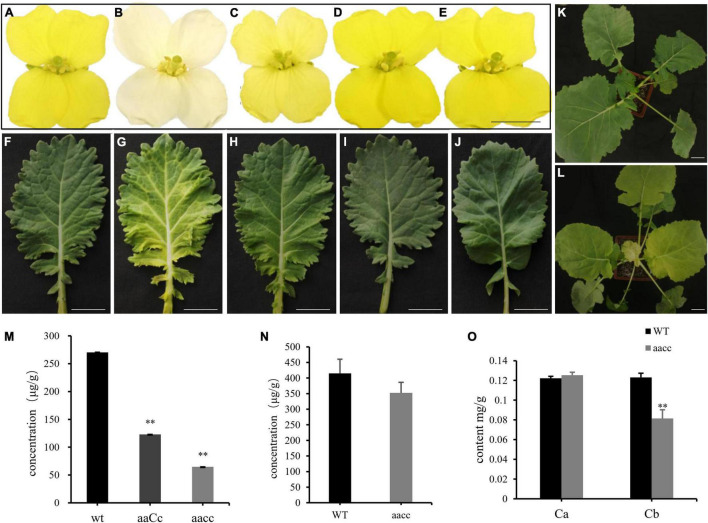
Mutations in the *BnaCRTISO* gene affected the color of petals and leaves. **(A–J)** The phenotype of flower (At the first day after flowering) and leaf in WT, *aacc*, *aaCc*, *AAcc*, *aaCC*, respectively. “*aaCC*,” “*AAcc*,” and “*aacc*” represent mutations of the target gene in *BnaA09.CRTISO*, *BnaC08.CRTISO* and both copies, respectively. **(K)** WT seeding. **(L)** *aacc* seeding. Bars, 1 cm. **(M)** The total carotenoid contents (μg/g fresh weight) of petals in aacc, aaCc, WT, respectively. **(N)** The total carotenoid contents of leaves in *aacc*, WT, respectively. **(O)** The content of chlorophyll *a*, *b* in the leaves of WT and *aacc*, respectively. **(M–O)** Data are presented as means ± SE (*n* ≥ 3); *t*-test was used for statistical analysis between the mutant and its WT (**P > 0.01).

To generate stable lines with targeted mutations, 72 independent T_0_ editing lines of *BnaCRTISO* were self-pollinated to produce T_1_, T_2,_ and T_3_ progenies. The targeted mutations of progenies from these T_0_ lines were verified by Hi-TOM sequencing analysis of the target sites (Table 1). The results proved that the mutant genotypes could be stably transmitted to the subsequent generations. A total of 10 T_3_ plants with homozygous mutations in *BnaCRTISO* were detected, including two *BnaA09.CRTISO* single mutants, two *BnaC08.CRTISO* single mutants and six *BnaCRTISO* double mutants (Table 1 and Figure 1C). These homozygous mutations at the target sites within *BnaCRTISO* were predicted to cause frameshifts resulting in the production of non-functional proteins (Supplementary Figure 6). As expected, all the double mutants could produce the creamy white flower phenotype (Figure 2B).

**TABLE 1:** Genotypic analysis of *BnaCRTISO* mutants and their transmission to T_1_, T_2_, and T_3_ generations.

Plant ID	Generation	Genotype at targets of *BnaCRTISO.A09*		Genotype at targets of *BnaCRTISO*.*C08*		Petal color
		S1	S2	S1	S2	
CRTISO-94-10	T1	WT	Homo (−2 bp)	Homo (−180 bp)		Creamy white
CRTISO-94-10-6	T2	WT	Homo (−2 bp)	Homo (−180 bp)		Creamy white
CRTISO-94-10-6-1	T3	WT	Homo (−2 bp)	Homo (−180 bp)		Creamy white
CRTISO-94-11	T1	WT	Biallelic	Homo (−180 bp)		Creamy white
CRTISO-94-12	T1	Hetero	Biallelic	Homo (−5 bp)	Homo (−1 bp)	Creamy white
CRTISO-94-13	T1	Hetero	Biallelic	Homo (−180 bp)		Creamy white
CRTISO-94-15	T1	Homo (+1 bp)	Homo (+1 bp)	Homo (−180 bp)		Creamy white
CRTISO-94-15-5	T2	Homo (+1 bp)	Homo (+ 1 bp)	Homo (−180 bp)		Creamy white
CRTISO-94-15-5-2	T3	Homo (+1 bp)	Homo (+1 bp)	Homo (−180 bp)		Creamy white
CRTISO-48-8	T1	Biallelic		WT	Biallelic (−9 bp, −1 bp)	Light yellow
CRTISO-48-9	T1	Homo (−166 bp)		Biallelic		Creamy white
CRTISO-48-10	T1	Homo (−166 bp)		WT	Homo (−9 bp)	Yellow
CRTISO-48-8-13	T2	WT	Homo (−1 bp)	WT	Homo (−1 bp)	Creamy white
CRTISO-48-8-13-1	T3	WT	Homo (−1 bp)	WT	Homo (−1 bp)	Creamy white
CRTISO-48-8-14	T2	WT	Hetero	Homo (−137 bp)		Yellow
CRTISO-48-8-15	T2	WT	Homo (−1 bp)	WT	Biallelic (−9, −1 bp)	Light yellow
CRTISO-48-8-18	T2	WT	Homo (−1 bp)	WT	Homo (−9 bp)	Yellow
CRTISO-48-10-4	T2	Homo (−166 bp)		WT	Homo (−1 bp)	Creamy white
CRTISO-48-10-4-1	T3	Homo (−166 bp)		WT	Homo (−1 bp)	Creamy white
CRTISO-48-8-20	T2	WT	Hetero	WT	Chimeric	Yellow
CRTISO-48-8-20-1	T3	WT	WT	WT	Homo (+ 1 bp)	Yellow
CRTISO-48-8-20-5	T3	WT	Homo (−1 bp)	WT	Biallelic (−9, −1 bp)	Light yellow
CRTISO-48-8-20-6	T3	WT	Homo (−1 bp)	WT	Homo (−9 bp)	Yellow
CRTISO-48-8-20-8	T3	WT	Homo (−1 bp)	WT	Homo (+ 1 bp)	Creamy white
CRTISO-48-8-21	T2	WT	Hetero	WT	Chimeric	Yellow
CRTISO-48-8-21-3	T3	WT	Homo (−1 bp)	WT	WT	Yellow
CRTISO-48-8-21-7	T3	WT	Hetero	WT	Homo (−9 bp)	Yellow
CRTISO-48-8-2	T2	WT	Homo (−1 bp)	WT	Biallelic (−9, −1 bp)	Light yellow
CRTISO-48-8-2-3	T3	WT	Homo (−1 bp)	WT	Biallelic (−9, −1 bp)	Light yellow
CRTISO-7-9	T1	Biallelic		Chimeric		Yellow
CRTISO-7-10	T1	Biallelic		WT	Homo (−5 bp)	Yellow
CRTISO-7-12	T1	WT	Homo (−6 bp)	WT	Homo (−5 bp)	Creamy white
CRTISO-7-18	T1	Biallelic		Homo (−180 bp)		Creamy white
CRTISO-7-2	T2	Chimeric		Biallelic		Yellow
CRTISO-7-2-2	T2	WT	Biallelic	WT	Homo (−5 bp)	Yellow
CRTISO-7-2-2-4	T3	WT	Homo (−19 bp)	WT	Homo (−5 bp)	Creamy white
CRTISO-7-2-2-7	T3	WT	Homo (−6 bp)	WT	Homo (−5 bp)	Yellow
CRTISO-7-6	T1	WT	Homo (−6 bp)	WT	Homo (+ 1 bp)	Yellow
CRTISO-7-6-1	T2	WT	Homo (−6 bp)	WT	Homo (+ 1 bp)	Yellow
CRTISO-7-6-1-1	T3	WT	Homo (−6 bp)	WT	Homo (+1 bp)	Yellow
CRTISO-43-2	T1	WT	Homo (−1 bp)	WT	Homo (−1 bp)	Creamy white
CRTISO-43-5	T1	Homo (−166 bp)		WT	Homo (−1 bp)	Creamy white
CRTISO-43-7	T1	WT	Homo (−1 bp)	WT	Homo (−9 bp)	Yellow
CRTISO-43-3	T1	Homo (−166 bp)		WT	Biallelic (−9, −1 bp)	Light yellow
CRTISO-43-3-4	T2	Homo (−166 bp)		WT	Biallelic (−9, −1 bp)	Light yellow
CRTISO-43-3-4-1	T3	Homo (−166 bp)		WT	Biallelic (−9, −1 bp)	Light yellow

*Hetero, heterozygous; Homo, homozygous; WT, wild type. “–” and “+” indicate the deletion and insertion of the indicated number of nucleotides or nucleotides, respectively; All other targets are wild type except the indicated target.*

The single mutants showed a similar yellow flower phenotype as WT (Figures 2A,D,E), while the heterozygous mutants (*aaCc*) showed a lighter yellow flower phenotype (Figure 2C). Thus, both copies of the *BnaCRTISO* gene function redundantly in regulating flower color. In addition, the inner leaves of the double mutants were also more yellowish than those of the WT, single mutants, and heterozygous mutants (Figures 2F–L), indicating that the *BnaCRTISO* gene also functions in the leaves.

### Off-Target Activity of CRISPR/Cas9 in T_0_ and T_1_ Transgenic *B. napus* Plants

To ascertain whether off-targeting occurred in the present study, we searched the *B. napus* genome for putative off-target sites with high homology to S1 and S2 according to the CRISPR-P program (Lei et al., 2014). A total of seven putative off-target sites were identified for both sgRNAs (Supplementary Table 10), and no off-target editing was detected in T_0_ and T_1_ by gene-specific primers amplification and Sanger sequencing (Supplementary Table 10). This result shows that the off-target effect is negligible when the specificity of each sgRNA is fully considered based on the genome sequence. Thus, the CRISPR/Cas9 system has a high specificity for targeted mutagenesis in *B. napus*.

### Subcellular Localization of *BnaCRTISO*

**FIGURE 3:**
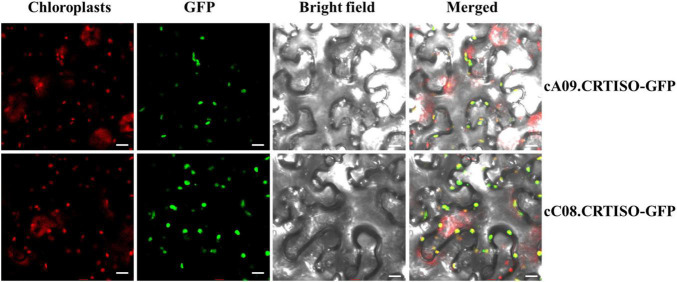
Subcellular localization of BnaA09.CRTISO and BnaC08.CRTISO in tobacco leaves. The fusion proteins generated using the coding sequence (cA09.CRTISO-GFP and cC08.CRTISO-GFP) were independently introduced into tobacco leaves. Scale bar = 10 μm.

In order to explore the subcellular localization of *BnaA09.CRTISO* and *BnaC08.CRTISO*, GFP was fused to the C terminal of each gene and transiently expressed in tobacco leaves. The green fluorescence signal overlaps closely with the chloroplast red autofluorescence signal observed by confocal microscopy (Figure 3). Thus, BnaCRTISO was predicted to be a chloroplast-localized protein, which was consistent with previous reports that carotenoids is synthesized and stored in plastids (Lange and Ghassemian, 2005).

### *BnaCRTISO* Regulates the Expression of Carotenes and Xanthophylls-Related Genes

The petals from flowers during anthesis were collected to compare the expression profiles between *BnaCRTISO* double mutants (CRTISO-94-15-5-2, CRTISO-48-8-13-1, CRTISO-7-2-2-4) and corresponding WT to investigate the transcriptome changes underlying the petal color variation (Supplementary Table 3). In subsequent analysis, a total of 41,186 genes were expressed in the developing petals during the same period. In the double mutants and WT, the pearson correlation coefficient between any two of the three biological repeats is very high (*R* = 0.93–0.98), indicating that the transcriptome sequencing data used in this study is highly reliable (Supplementary Figure 7).

Comparing the transcript abundance in these petals, it was observed that there were 2,058 DEGs between the *BnaCRTISO* double mutants and its corresponding WT at the same period (Supplementary Table 4). Overall, 953 genes were up-regulated in the double mutant petals, while 1,105 genes were down-regulated, which may be related to the changes observed in petal color (Supplementary Figure 8). The GO and KEGG enrichment analysis of these identified DEGs showed that the metabolic processes of carotenoid and flavonoid were significantly enriched among the down-regulated DEGs in mutants relative to WT (Supplementary Figures 9–11 and Supplementary Tables 5–7).

**FIGURE 4:**
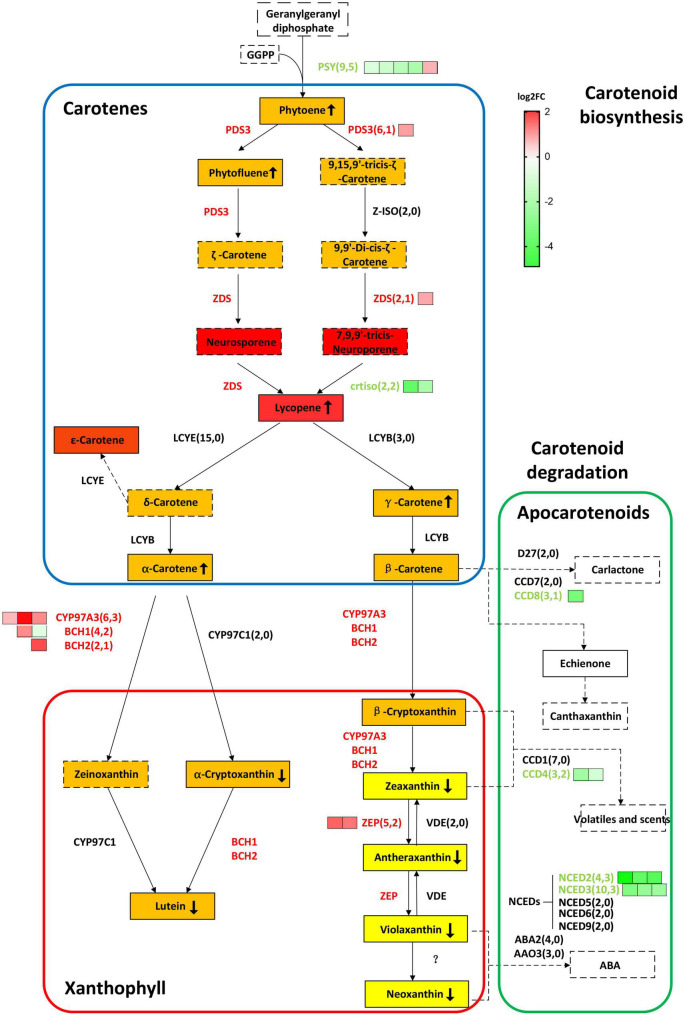
*BnaCRTISO* regulates genes related to carotenes and xanthophyll in rapeseed. In this study, the gene copy numbers from the rapeseed genome and DEGs are listed in parentheses. The log_2_ fold change (*crtiso*/WT) of DEGs of different copies of the same gene is represented by green (down-regulated) or red (up-regulated) squares arranged from left to right. Gene names are represented in capital letters, and corresponding mutants are represented in small letters. Genes that are up-regulated and down-regulated are represented in red and blue, respectively. The background color of the box indicates the color of the substance. The detected carotenoids in present study were indicated in the solid box, and undetected carotenoids in present study were indicated in the dotted box. The up or down arrow in the box indicates increase or decrease of the corresponding substance content, respectively. PSY, PHYTOENE SYNTHASE; PDS3, 15-*cis*-zeta-carotene isomerase; Z-ISO, 15-*cis*-zeta-carotene isomerase; ZDS, zeta-carotene desaturase; CRTISO, carotenoid isomerase; LCYE, lycopene beta/epsilon cyclase protein; LCYB, lycopene cyclase; BCH1, beta-hydroxylase 1; BCH2, beta-carotene hydroxylase 2; CYP97A3, cytochrome P450, family 97, subfamily A, polypeptide 3; CYP97C1, cytochrome P450 superfamily protein; ZEP, zeaxanthin epoxidase; VDE, non-photochemical quenching 1; NCED5,nine-*cis*-epoxycarotenoid dioxygenase 5; NCED3, nine-*cis*-epoxycarotenoid dioxygenase 3; NCED6, nine-*cis*-epoxycarotenoid dioxygenase 6; NCED9, nine-*cis-*epoxycarotenoid dioxygenase 9; NCED2, nine-*cis-*epoxycarotenoid dioxygenase 2; CCD4, nine-*cis-*epoxycarotenoid dioxygenase 4; ABA2, NAD(P)-binding Rossmann-fold superfamily protein; AAO3, abscisic aldehyde oxidase 3; CYP707A2, cytochrome P450, family 707, subfamily A, polypeptide 2; CYP707A4, cytochrome P450, family 707, subfamily A, polypeptide 4; CYP707A3, cytochrome P450, family 707, subfamily A, polypeptide 3; CYP707A1, cytochrome P450, family 707, subfamily A, polypeptide 1; D27, beta-carotene isomerase D27-like protein; CCD7, carotenoid cleavage dioxygenase 7; CCD8, carotenoid cleavage dioxygenase 8.

As the targeted mutated gene, *BnaC08.CRTISO* and *BnaA09.CRTISO* were down-regulated by almost eight and three times, respectively. The expression levels of most carotenoid biosynthesis-related genes were significantly up-regulated, such as *BnaPDS3*, *BnaZDS*, *BnaCYP97A3*, *BnaBCH1*, *BnaBCH1*, *BnaZEP*; whereas, five copies of *BnaPSY* were significantly down-regulated. *BnaPSY*, *BnaPDS3*, *BnaZDS*, and *BnaCRTISO* are involved in carotene synthesis; *BnaCYP97A3*, *BnaBCH1 BnaBCH1*, and *BnaZEP* are related to xanthophylls synthesis. *PSY*, encoding a phytoene synthase, acts like a faucet and plays a significant role at the initial stage in the carotenoid biosynthesis pathway. Moreover, some genes in the carotenoid degradation pathway were also significantly down-regulated, including *BnaCCD4*, *BnaCCD8*, *BnaNCED2*, and *BnaNCED3* (Figure 4 and Supplementary Table 7).

To verify the reliability of the RNA-seq data, 29 DEGs in the petals were selected for qRT-PCR verification analysis. These DEGs include 13 genes involved in the carotenoid synthesis, five genes involved in the flavonoid metabolism, and 11 randomly selected genes. Linear regression analysis showed that the correlation coefficient between the transcript levels assessed by the two analytic systems was very high (*R* = 0.812; Supplementary Figure 12), which further confirmed the reliability of the RNA-seq data.

Together, these results indicated the importance of the *BnaCRTISO* gene in the metabolic pathway of carotenoid. This also further illustrates the complex regulatory mechanisms of the *BnaCRTISO* gene in carotenoid synthesis of rapeseed.

### Targeted Mutations in *BnaCRTISO* Change Pigment Concentrations

To assess the impact of targeted mutation of *BnaCRTISO* on the carotenoid metabolic pathway, double (*aacc*: CRTISO-94-15-5-2, CRTISO-48-8-13-1, CRTISO-7-2-2-4) and heterozygous (*aaCc*: CRTISO-43-3-4-1, CRTISO-48-8-2-3, CRTISO-48-8-20-5) mutant T_3_ lines were grown in the field with their WT control. The total carotenoid content of petal and leaf samples were analyzed using spectrophotometer. It showed that the total carotenoid content of petals were significantly decreased in *aacc* mutants compared with *aaCc* and WT (*aacc* < *aaCc* < WT) (Figure 2M). The similar trend was observed in the total carotenoid content of leaves (Figure 2N). The two types of chlorophyll (chlorophyll *a* and chlorophyll *b*) concentration were further measured in leaves. And the chlorophyll *b* concentration of *aacc* mutants were significantly decreased relative to WT; whereas, no difference in chlorophyll *a* concentrations were observed between *aacc* mutants and WT (Figure 2O). This result indicated that *BnaCRTISO* may be involved in the anabolism of chlorophyll *b* in leaves.

**FIGURE 5:**
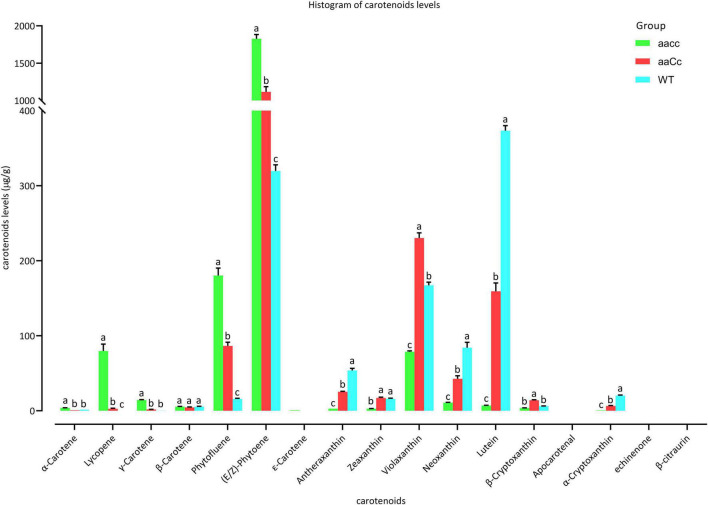
The different carotenoids levels in the petals of mutants and WT. *aacc*, homozygous double mutants; *aaCc*, the heterozygous mutants with a lighter yellow flower phenotype; WT, wild type; LSD-test was used for statistical analysis between the aacc and WT (Lower case letter indicates the significant difference at 0.05 level).

Furthermore, carotenoid were analyzed using an LC-MS/MS system. Almost all carotene and xanthophyll contents showed a significant difference among *aacc*, *aaCc*, and WT. The carotene content of *aacc* was significantly increased when compared to the *aaCc* mutant and WT (*aacc* > *aaCc* > WT); while the xanthophyll content was significantly decreased (*aacc* < *aaCc* < WT) (Figure 5 and Supplementary Table 8). (E/Z)-phytoene, phytofluene, and lycopene are the main components of carotene. In this study, no lycopene was detected in the petals of WT; unexpectedly, the lycopene contents of the *aacc* and *aaCc* mutant were significantly increased (*aacc* > *aaCc* > WT; Supplementary Table 8). It should be noted that lycopene produced by the BnaCRTISO enzyme catalyzes prolycopene conversion in the carotenoid pathway. This result indicates the occurrence of another pathway for lycopene biosynthesis or degradation in the *BnaCRTISO* mutant, which converts prolycopene into all-*trans* lycopene through a non-enzymatic reaction under light illumination (Tuan et al., 2011; Su et al., 2015). In the xanthophyll metabolic pathway, the contents of α-cryptoxanthin (synthetic lutein precursor substance), β-cryptoxanthin (zeaxanthin precursor substance), lutein, zeaxanthin, antheraxanthin, violaxanthin, and neoxanthin were all significantly decreased in *aacc* mutants (Figure 5 and Supplementary Table 8). These changes of xanthophylls content agreed well with the phenotypic variations in these materials: the higher the xanthophylls content, the yellower the petals.

### Targeted Mutations in *BnaCRTISO* Change Flavonoid Metabolites in Petals

**FIGURE 6:**
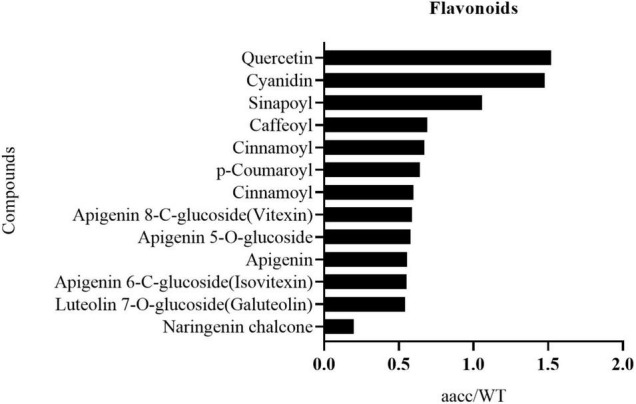
The identified flavonoid metabolites in petals with significant difference between the *BnaCRTISO* double mutant (*aacc*) and WT. The level of most of these metabolites decreased in mutant petals. And they are listed in the order of decreased fold changes (*aacc*/WT) from top to down.

The flavonoid metabolic profiling of petal samples from the double-mutant (CRTISO-94-15-5-2, CRTISO-48-8-13-1, CRTISO-7-2-2-4) and its WT was analyzed using LC-ESI-MS/MS system. Most of the identified flavonoid metabolites show a significant difference between the double-mutant and WT petals (Supplementary Table 8). Among them, naringenin chalcone, the main flavonoid metabolites that constitutes yellow petals, were significantly decreased in the mutant. It is consitent with the transcriptomic analysis of the mutant petals, which indicates that the expression of naringenin chalcone metabolic genes (*BnaC4H*, *BnaTT4*, *BnaTT7*, *BnaPAL2*, *Bna4CL3*) were decreased in the double mutant (Figure 6, Supplementary Figure 12, and Supplementary Table 9). The content of other anthocyanins (apigenin and luteolin) in the mutants also decreased significantly (Figure 6 and Supplementary Table 9). Thus, these results are in line with the phenotypes and the transcriptomic analysis of the mutant petal.

## Discussion

### The CRISPR/Cas9-Targeted Mutation in *BnaCRTISO* Is a Promising Strategy to Change Flower Color in Rapeseed

Although flower color in *B. napus* is a very important trait and has attracted much interest since 1929 (Pearson, 1929), the underlying molecular mechanisms have not been well characterized. The white flower is an ornamental trait and ideal phenotypic marker in assisted breeding. Studies has also indicated that the white flower trait of *B. napus* is closely linked with high erucic acid (Liu et al., 2004). Therefore, it is significant to develop phenotypic markers and improve the ornamental value of low erucic acid rapeseed. To this end, an effective approach is needed to produce targeted mutations in these well-conserved *CRTISO* homologs in *B. napus*. The newly developed CRISPR/Cas9 technology provides a powerful approach for creating novel allelic variations. Thus far, it has been successfully utilized to modify several important agronomic traits (multilocular silique, plant height, architecture, pod shatters resistance, etc.) in rapeseed by generating specific gene knockout (Yang et al., 2017, 2018; Li C. et al., 2018; Zhai et al., 2019).

This study shows the successful utilization of CRISPR/Cas9 for targeted mutations of the *BnaCRTISO* gene in rapeseed with an editing efficiency of 75.00%, which is higher than those reported in previous research (14.4–45.0%) (Yang et al., 2017; Zhai et al., 2019; Ahmar et al., 2021). The editing efficiency at the S2 target site (73.22%) is higher than S1 (16.39%), indicating that the efficiency of the sgRNA promoter U3b is higher than that of U3d as our previous reports (Yang et al., 2017; Zhai et al., 2019; Ahmar et al., 2021). In this study, the targeted mutations of the two functional copies of *BnaCRTISO* in rapeseed generated the creamy white petal phenotype (Supplementary Table 2 and Figure 2), indicating that the *BnaCRTISO* gene is essential for flower color regulation and highly conservative in *Brassica* plants. The phenotypic analysis shows that the flower color of the double mutants is creamy white, while the *BnaA09.CRTISO* and *BnaC08.CRTISO* single mutants showed a comparable phenotype to that of the WT. Hence, both copies of the *BnaCRTISO* gene have functional redundancy in regulating flower color formation, and their contribution is equal. In addition, the heterozygous mutant (*aaCc*) identified in this study had a biallelic mutation (−9, −1 bp; e.g., CRTISO-8-15) on the *BnaC08.CRTISO* with “aa” genotype, and the plant shows a slight change in petal color (Figure 2B). While the double allelic mutation (−6, −19 bp; e.g., CRTISO-7-2-2) on *BnaA09.CRTISO* with “cc” genotype shows no change in petal color (Table 1). A homozygous −9 bp mutation on *BnaC08.CRTISO* with “aa” genotype (CRTISO-48-10, CRTISO-48-8-18, CRTISO-48-8-20-6, CRTISO-48-8-21-7, CRTISO-43-7) also shows no change in petal color (Table 1). Thus, these results indicated that different mutation types have different effects on BnaCRTISO enzyme activity.

### *BnaCRTISO* Plays an Important Role in the Accumulation of Pigments in Petals

Flavonoids and carotenoids are the pivotal pigments for the formation of most flower colors (Zhang et al., 2015). It has been reported that *BnaC3.CCD4* and *CmCCD4a* contribute to the white color formation in the petals of *B. napus* and *chrysanthemum*, respectively, by degrading carotenoids into colorless compounds (Ohmiya et al., 2006; Zhang et al., 2015). Recently, Zhao et al. (2021) identified a mutation in a *PDS3* gene in carotenoid biosynthesis pathway causes yellowish-white petals in rapeseed (Zhao et al., 2021). To date, no gene regulating the white flower traits other than the *CCD4* and *PDS3* gene has been reported in rapeseed.

In *Arabidopsis*, the *crtiso* mutant exhibiting partial inhibition of lutein biosynthesis in light-grown tissue and the accumulation of poly-*cis*-carotene precursors in dark-grown tissue with cotyledons colors changing from yellow to orange (Hyoungshin, 2002). The loss of function of the *BrCRTISO* gene confers orange color to the inner leaves and induces changes in flower color from yellow to orange in Chinese cabbage (*B. rapa*) (Su et al., 2015). Recently, the targeted editing of *BoaCRTISO* changed the leaf color from green to yellow, with the significant reduction of the concentrations of carotenoids and chlorophylls in Chinese kale (Sun et al., 2020). The *crtiso* mutant in tomato accumulates prolycopene instead of all-*trans*-lycopene, which could result in the production of orange fruit (Kato et al., 2007). It shows that the *CRTISO* gene in different plants can effectively change the carotenoid content in the flower, leaf, or fruit. In rapeseed, the cotyledon color of the *BnaCRTISO* double mutant showed a similar color change as that in *B. rapa* under the dark treatment (Supplementary Figure 13). Additionally, the petal color of the *BnaCRTISO* mutant changed from yellow to creamy white, which is different from that of *B. rapa* and *Arabidopsis*. It implies that the function of *CRTISO* in allotetraploid *B. napus* has become more complex in the course of evolution, and more information about this gene involved in the carotenoid pathway is yet to be ascertained.

PSY is the first key enzyme in the synthesis pathway, and its expression is regulated by feedback from upstream and downstream genes and metabolites (Yonekura-Sakakibara et al., 2019). The *BnaPSY* gene is also an important gene encoding the rate-limiting enzyme in the carotenoid synthesis pathway (Zhou et al., 2015). Down-regulation of the *BnaPSY* gene leads to a decrease in the total carotenoids content in the petals of the *BnaCRTISO* double mutants. Moreover, the xanthophyll content also decreased drastically in the *BnaCRTISO* mutants, and there are almost no xanthophylls in the petals of the double mutants. In contrast, the content of lycopene synthesized by the catalysis of BnaCRTISO protein increased significantly in the *BnaCRTISO* mutant. This result is consistent with the changes in the carotenoids content measured in the leaves of *Arabidopsis CRTISO* mutants (Isaacson, 2002). The expression of key genes *BnaPDS3* and *BnaZDS* in the carotenoid synthesis pathway was also significantly up-regulated. This shows that the steady down-regulation of *BnaCRTISO* resulted in the up-regulation of *BnaPDS3* and *BnaZDS*, which promote the accumulation of prolycopene and the mass synthesis of lycopene *via* a non-enzymatic pathway (Figure 4).

The expression levels of several xanthophyll synthetic-related genes (*BnaCYP97A3*, *BnaBCH1*, *BnaBCH2*, *BnaZEP*) in the mutants are up-regulated. Compared with the slight change in the synthetic substrate content, the content of the downstream substances (α-cryptoxanthin, lutein, zeaxanthin, antheraxanthin, violaxanthin, neoxanthin) are decreased significantly (Figure 4 and Supplementary Table 8). Interestingly, the expression of genes that inhibit ABA hormone signal transduction, such as *BnaHAI1*, *BnaABI1*, and *BnaHAI2*, are significantly down-regulated (Supplementary Table 7). In contrast, the expression of the ABA hormone signaling receptor *BnaPYL4* is significantly up-regulated (Supplementary Table 7). It shows that the ABA hormone signal transduction process is accelerated in the mutant. Previoly reports indicated that ABA is synthesized from xanthophylls, e.g., zeaxanthin, violaxanthin and neoxanthin (Li and Walton, 1990; Parry et al., 1990). Thus, the low content of xanthophylls in the *BnaCRTISO* mutant is probably due to its degradation.

In the past, researchers created various types of light-colored flowers in different plants by changing the expression of the *CHS* gene to generate either white or light white flowers (Wang et al., 2018). In this study, the expression of multiple copies of the *BnaCHS* gene, which is the upstream gene regulating the flavonoid metabolism, was significantly down-regulated in the *BnaCRTISO* mutant resulting in the closure of the entire flavonoid synthesis pathway and a sharp decrease in the flavonoids content in petal (Supplementary Table 7). *BnaF3′H* is a key gene regulating for anthocyanin synthesis in the flavonoid synthesis pathway (Dubos et al., 2010; He et al., 2013), and its expression in the mutant is also greatly down-regulated. Collectively, these results indicate that the *BnaCRTISO* is an important gene involved in both the carotenoid and flavonoid pathways.

At present, there is no direct correlation between the carotenoid pathway and flavonoid synthesis pathway in plants (Grotewold, 2006; Cazzonelli and Pogson, 2010). It is known that they jointly regulate the color of flowers through the accumulation and mixing of pigments to produce their colored substances. In this study, a creamy white flower mutant was obtained by the mutation of the *BnaCRTISO* gene. Through metabolome and transcriptome analysis, we observed that the loss of *BnaCRTISO* gene function affects not only the expression of related genes in the carotenoid pathway, but also the expression of key genes involved in the flavonoid synthesis pathway. This reveals that some unknown mechanism of interactions exists between the carotenoid and flavonoid pathway in *B. napus* that are worthy of further study.

## Data Availability Statement

The datasets presented in this study can be found in online repositories. The names of the repository/repositories and accession number(s) can be found in the article/Supplementary Material.

## Author Contributions

YZ and CF conceived the study and designed the experiments. LH performed the experiments. KY performed the bioinformatic analysis and wrote the manuscript. YY, ML, MG, and SD helped in the material sampling. LH, OA, and CF helped in the revision of this manuscript. CF supervised the study. All authors contributed to the article and approved the submitted version.

## Conflict of Interest

The authors declare that the research was conducted in the absence of any commercial or financial relationships that could be construed as a potential conflict of interest.

## Publisher’s Note

All claims expressed in this article are solely those of the authors and do not necessarily represent those of their affiliated organizations, or those of the publisher, the editors and the reviewers. Any product that may be evaluated in this article, or claim that may be made by its manufacturer, is not guaranteed or endorsed by the publisher.

## Footnotes

1
http://www.hi-tom.net/hi-tom/

2
http://crispr.hzau.edu.cn/CRISPR2/
