# Supplementary material for: Site-Directed Mutagenesis of the Carotenoid Isomerase Gene BnaCRTISO Alters the Color of Petals and Leaves in Brassica napus L
Source: Front Plant Sci. 2022 Feb 10;13:801456. doi: 10.3389/fpls.2022.801456 (PMC8866652; doi:10.3389/fpls.2022.801456)
Supplement: Supplementary file 1 [file Data_Sheet_1.docx]

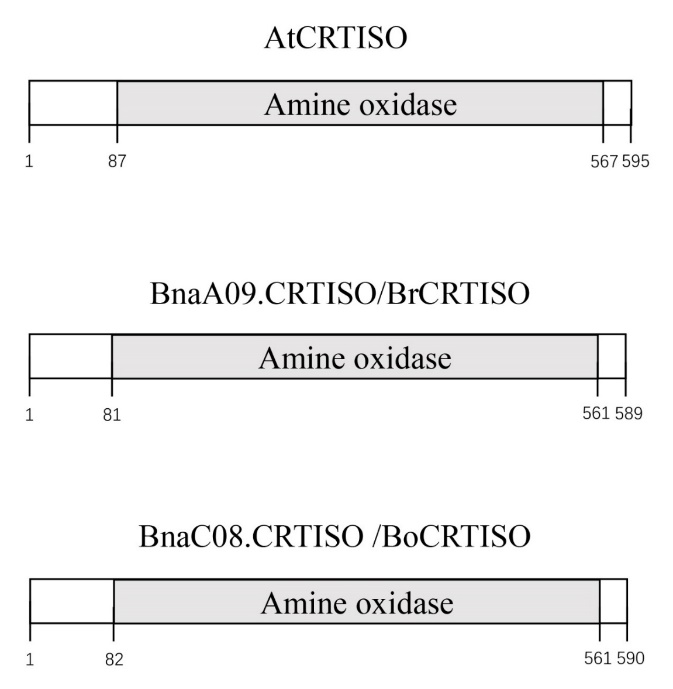


**Fig. S1 Organization of the predicted CRTISO protein indicating the localization of the conserved domains in *Arabidopsis* and different *Brassica* species.** Protein sequences are obtained from GenBank with the following accession numbers: AtCRTISO (Q9M9Y8) in *Arabidopsis thaliana*; BnaA09.CRTISO ([XP_013716452.1](https://www.ncbi.nlm.nih.gov/protein/923890672)) and BnaC08.CRTISO(CDY09954.1) in *B. napus;* BoCRTISO(A0A0D3DYP6) in *B. oleracea*; BrCRTISO (VDC64404.1) in *B. rapa*.

AtCRTISO MDLCFQNPVKCGDRL---FSALNTSTYYKLGTSNLGFNGPVLENRKKKKKLPRMVTVKSV 57

BjuCRTISO MNLCLHNPVACADRSSSLFSALKT-SNYKLGTSKFGFLKHRK--------KNHVVAVRSV 51

BrCRTISO MNLCLHNPVTCADRSSSLFSALKT-SNNKLGTSKFGFLKNRK--------KNHVVAVRSV 51

BnaA09.CRTISO MNLCLHNPVTCADRSSSLFSALKT-SNNKLGTSKFGFLKNRK--------KNHVVAVRSV 51

BnaC08.CRTISO MNLCLRNPVTCADRSSSLFSALKT-SNNKLGTSKFGFLKNPK--------KNHVVAVRSV 51

BoCRTISO MNLCLRNPVTCADRSSSLFSALKT-SNYKLGTSKFGFLKHHK--------KNHVVTVRYV 51

AtCRTISO SSSVVAST-VQGTKRDGGESLYDAIVIGSGIGGLVAATQLAVKEARVLVLEKYLIPGGSS 116

BjuCRTISO STSTVTT---VKEETKRESQVYDAIVIGSGIGGLVAATQLAVKEAKVLVLEKYLIPGGSS 108

BrCRTISO SSTA-VEERTKRESGGGESKVYDAIVIGSGIGGLVAATQLAVKEAKVLVLEKYLIPGGSS 110

BnaA09.CRTISO SSTA-VEERTKRESGGGESKVYDAIVIGSGIGGLVAATQLAVKEAKVLVLEKYLIPGGSS 110

BnaC08.CRTISO SSTAVVEERTKRESGGGESKVYDAIVIGSGIGGLVAATQLAVKEAKVLVLEKYLIPGGSS 111

BoCRTISO SSTAVVEERTKRENGGGESKVYDAIVIGSGIGGLVAATQLSVKEAKVLVLEKYLIPGGSS 111

AtCRTISO GFYERDGYTFDVGSSVMFGFSDKGNLNLITQALKAVGRKMEVIPDPTTVHFHLPNNLSVR 176

BjuCRTISO GYYERDGYTFDVGSSVMFGFSDKGKLNLITQALKAVGREMEVIPDPTTVHFHLPNDLSVQ 168

BrCRTISO GYYERDGYTFDVGSSVMFGFSDKGNLNLITQALKAVGREMEVIPDPTTVHFHLPNDLSVQ 170

BnaA09.CRTISO GYYERDGYTFDVGSSVMFGFSDKGKLNLITQALKAVGREMEVIPDPTTVHFHLPNDLSVQ 170

BnaC08.CRTISO GYYERDGYTFDVGSSVMFGFSDKGNLNLITQALKAVGREMEVIPDPTTVHFHLPSDLSVQ 171

BoCRTISO GYYERDGYTFDVGSSVMFGFSDKGNLNLITQALKAVGREMEVIPDPTTVHFHLPSDLAVQ 171

AtCRTISO IHREYDDFIAELTSKFPHEKEGILGFYGDCWKIFNSLNSLELKSLEEPIYLFGQFFQKPL 236

BjuCRTISO VHREYDEFVNELISKFPHEKEGILGFYGICWKIFNSLNSLELKSLEEPIYLFGQFFQKPL 228

BrCRTISO VHREYDEFVNELISKFPHEKEGILGFYGICWKIFNSLNSLELKSLEEPIYLFGQFFQKPL 230

BnaA09.CRTISO VHREYDEFVNELISKFPHEKEGILGFYGVCWKIFNSLNSLELKSLEEPIYLFGQFFQKPL 230

BnaC08.CRTISO VHREYDEFVNELISKFPHEKEGILGFYGVCWKIFNSLNSLELKSLEEPIYLFGQFFQKPL 231

BoCRTISO VHREYDEFVNELISKFPHEKDGILGFYGVCWKIFNSLNSLELKSLEEPIYLFGQFFQKPL 231

AtCRTISO ECLTLAYYLPQNAGAIARKYIKDPQLLSFIDAECFIVSTVNALQTPMINASMVLCDRHYG 296

BjuCRTISO ECLTLAYYLPQNAGDIARKYIKDPQLLSFIDAECFIVSTVNALQTPMINASMVLCDRHYG 288

BrCRTISO ECLTLAYYLPQNAGDIARKYIKDPQLLSFIDAECFIVSTVNALQTPMINASMVLCDRHYG 290

BnaA09.CRTISO ECLTLAYYLPQNAGDIARKYIKDPQLLSFIDAECFIVSTVNALQTPMINASMVLCDRHYG 290

BnaC08.CRTISO ECLTLAYYLPQNAGDIARKYIKDPQLLSFIDAECFIVSTVNALQTPMINASMVLCDRHYG 291

BoCRTISO ECLTLAYYLPQNAGDIARKYIKDPQLLSFIDAECFIVSTVNALQTPMINASMVLCDRHYG 291

AtCRTISO GINYPVGGVGGIAKSLAEGLVDQGSEIQYKANVKSIILDHGKAVGVRLADGREFFAKTII 356

BjuCRTISO GINYPVGGVGGIARSLAGGLVDQGSEILYKANVKRIILDDGKAVGVRLADGREFYAKTII 348

BrCRTISO GINYPVGGVGGIARSLAGGLVDQGSEILYKANVKSIILDDGKAVGVRLADGREFFAKTII 350

BnaA09.CRTISO GINYPVGGVGGIARSLAGGLVDQGSEILYKANVKSIILDDGKAVGVRLADGREFFAKTII 350

BnaC08.CRTISO GINYPVGGVGGIAKSLADGLVDQGSEILYKANVKSIILDDGKAVGVRLADGREFFAKTII 351

BoCRTISO GINYPVGGVGGIAKSLAEGLVDQGSEILYKANVKSIILDDGKAVGVRLADGREFFAKTII 351

AtCRTISO SNATRWDTFGKLLKGEKLPKEEENFQKVYVKAPSFLSIHMGVKAEVLPPDTDCHHFVLED 416

BjuCRTISO SNATRWDTFGKLLKGEKLPKEEENFQKVYVKAPSFLSIHMGVKAEVLPPDTDCHHFVLED 408

BrCRTISO SNATRWDTFGKLLKGEKLPKEEENFQKVYVKAPSFLSIHMGVKAEVLPPDTDCHHFVLED 410

BnaA09.CRTISO SNATRWDTFGKLLKGEKLPKEEENFQKVYVKAPSFLSIHMGVKAEVLPPDTDCHHFVLED 410

BnaC08.CRTISO SNATRWDTFGKLLKGEKLPKEEENFQKVYVKAPSFLSIHMGVKAEVLPPDTDCHHFVLED 411

BoCRTISO SNATRWDTFGKLLKGEKLPKEEENFQKVYVKAPSFLSIHMGVKAEVLPPDTDCHHFVLED 411

AtCRTISO DWKNLEEPYGSIFLSIPTILDSSLAPDGRHILHIFTTSSIEDWEGLPPKEYEAKKEDVAA 476

BjuCRTISO DWKNLEEPYGSIFLSIPTILDPSLAPDGRHILHIFTTSSIEDWEGLTPKEYEAKKEEVAA 468

BrCRTISO DWKNLEEPYGSIFLSIPTILDPSLAPDGRHILHIFTTSSIEDWEGLTPKEYEAKKEEVAA 470

BnaA09.CRTISO DWKNLEEPYGSIFLSIPTILDPSLAPDGRHILHIFTTSSIEDWEGLTPKEYEAKKEEVAA 470

BnaC08.CRTISO DWKNLEEPYGSIFLSIPTILDPYLAPDGRHILHIFTTSSIEDWEGLTPKEYEAKKEEVAA 471

BoCRTISO DWKNLEEPYGSIFLSIPTILDPYLAPDGRHILHIFTTSSIEDWEGLTPKEYEAKKEEVAA 471

AtCRTISO RIIQRLEKKLFPGLSSSITFKEVGTPRTHRRFLARDKGTYGPMPRGTPKGLLGMPFNTTA 536

BjuCRTISO GIIQRLEKKLFPGLSSSITFKEVGTPRTHRRYLARDKGTYGPMPRGTPKGLLGMPFNTTA 528

BrCRTISO GIIQRLEKKLFPGLSSSITFKEVGTPRTHRRYLARDKGTYGPMPRGTPKGLLGMPFNTTA 530

BnaA09.CRTISO GIIQRLEKKLFPGLSSSITFKEVGTPRTHRRYLARDKGTYGPMPRGTPKGLLGMPFNTTA 530

BnaC08.CRTISO GIIQRLEKKLFPGLSSSITFKEVGTPRTHRRYLARDKGTYGPMPRGTPKGLLGMPFNTTA 531

BoCRTISO GIIQRLEKKLFPGLSSSITFKEVGTPRTHRRYLARDKGTYGPMPRGTPKGLLGMPFNTTA 531

AtCRTISO IDGLYCVGDSCFPGQGVIAVAFSGVMCAHRVAADIGLEKKSRVLDVGLLGLLGWLRTLA 595

BjuCRTISO IDGLYCVGDSCFPGQGVIAVAFSGVMCAHRVAADIGLERKSKVLDAGLLGLLGWLRTLA 587

BrCRTISO IDGLYCVGDSCFPGQGVIAVAFSGVMCAHRVAADIGLERKSKVLDAGLLGLLGWLRTLA 589

BnaA09.CRTISO IDGLYCVGDSCFPGQGVIAVAFSGVMCAHRVAADIGLERKSKVLDAGLLGLLGWLRTLA 589

BnaC08.CRTISO IDGLYCVGDSCFPGQGVIAVAFSGVMCAHRVAADIGLERKSKVLDAGLLGLLGWLRTLA 590

BoCRTISO IDGLYCVGDSCFPGQGVIAVAFSGVMCAHRVAADIGLERKSKVLDAGLLGLLGWLRTLA 590

**Fig. S2** Alignment of CRTISO homolog sequences identified from *B. napus* (BnaA09.CRTISO and BnaC08.CRTISO), *B. rapa* (BrCRTISO), *B. oleracea* (BoCRTISO), *B. juncea* (BjuCRTISO), and *A. thaliana* (AtCRTISO). The Amine oxidase domain is highlighted in grey boxes.

BnaA09.CRTISO -----TTTTTTTCTCTTTTTTTTTTGTAATATCCTGCACTTCTGAGTGTTCTTCACCAAA 55

BnaC08.CRTISO TTTTTTTTTTCTTTTTTTTTTTTTTTTAATATCCTGCACTTGCGAGTGTTCTTCACCAAA 60

***** * * ********** *************** *****************

BnaA09.CRTISO CATTTTTTTGTCCTTCCATGTGATGAAAACCAATTTTCCAAATTCGGATGAAAGGACAAC 115

BnaC08.CRTISO CATTT-TTTTTCCTTCCACATGATGAAAATCAATTTTCCAAATTCGGATGAAAAGACAAC 119

***** *** ******** ********* *********************** ******

BnaA09.CRTISO ACTTTTTAAATCTGTCTGAAGAAGAACTGATTAGGTTTGGTACTATCCAAAGGACACAAC 175

BnaC08.CRTISO ACTTTTTAAGTCTGTCTGATGGAGAATTGATTAGGTTCCGTAATATCCAAAGGACACAAC 179

********* ********* * **** ********** *** *****************

S1

BnaA09.CRTISO ACAGTGACGACGAACCAAGCTCAGAACCATGAATCTCTGTCTCCACAATCCCGTAACGTG 235

BnaC08.CRTISO ACAGTGACGACGAACCAAGCTCAGAGCCATGAATCTCTGTCTCCGCAACCCCGTAACGTG 239

************************* ****************** *** ***********

BnaA09.CRTISO TGCTGATCGCAGCTCCTCCTTGTTCTCCGCCTTGAAGACTTCAAATAACAAGTTGGGTAC 295

BnaC08.CRTISO TGCTGATCGCAGCTCCTCCTTGTTCTCGGCCTTGAAGACTTCAAATAACAAGCTGGGTAC 299

*************************** ************************ *******

BnaA09.CRTISO TTCAAAGTTTGGGTTTTTAAAGAATCGGAAGAAGAATCATGTGGTTGCAGTGAGATCTGT 355

BnaC08.CRTISO TTCAAAGTTTGGGTTTTTAAAGAATCCGAAGAAGAATCATGTGGTTGCAGTGAGGTCTGT 359

************************** *************************** *****

S2

BnaA09.CRTISO TTCTTCCACTGCTGTAG---AAGAAAGGACGAAGAGAGAAAGTGGAGGAGGAGAGAGTAA 412

BnaC08.CRTISO TTCTTCCACTGCTGTAGTAGAAGAAAGGACGAAGAGAGAAAGTGGAGGAGGAGAGAGTAA 419

***************** ****************************************

BnaA09.CRTISO AGTGTACGACGCAATCGTCATCGGGTCTGGGATTGGAGGATTAGTTGCGGCGACTCAGCT 472

BnaC08.CRTISO AGTATACGACGCAATCGTCATCGGGTCTGGGATAGGAGGCTTAGTTGCGGCGACTCAGCT 479

*** ***************************** ***** ********************

BnaA09.CRTISO AGCTGTTAAAGAAGCTAAAGTTTTAGTTTTGGAGAAGTATCTGATCCCTGGTGGGAGCTC 532

BnaC08.CRTISO AGCTGTTAAAGAAGCTAAAGTTCTAGTTTTGGAGAAGTATCTCATCCCTGGTGGGAGCTC 539

********************** ******************* *****************

BnaA09.CRTISO CGGTTATTACGAAAGAGATGGATACACATTCGATGTTGGCTCTTCTGTCATGTTTGGTTT 592

BnaC08.CRTISO TGGTTATTACGAAAGAGATGGGTACACATTCGATGTGGGTTCCTCTGTCATGTTTGGTTT 599

******************** ************** ** ** *****************

BnaA09.CRTISO CAGCGATAAGGTTTGTTTCGTTTGCTCTCGTAAAGACTCTCCCTTTAGATGAGAATGTTG 652

BnaC08.CRTISO CAGCGATAAGGTTAG-CTCGTTTGCTCTCGTAAAGACTCTCCCTTTAGATGAGAATGTTG 658

************* * *******************************************

BnaA09.CRTISO TGTCACCATCAT----CTCTATTGAAAAATAAAAATAAGTAAATTACTCTATTTATATAG 708

BnaC08.CRTISO AGTCACCCACCATCATCTCTATTGAAAAATAAAAATAAGTAAATTACTCTATTTATA--- 715

****** * *****************************************

BnaA09.CRTISO TAAATCAATTTTTAACTCATTTATAGAGTGAAACTAGAGTAATATTAGAGCATTTTTACT 768

BnaC08.CRTISO ------------------------------------------------------------ 715

BnaA09.CRTISO CTAAAAATAGAGTGTGGTCGGAGATGGACTAATGAGTTTAGTTTTGATGGGGACAGGGGA 828

BnaC08.CRTISO --TAGCATAAAGCAGGGTTGGAGATGCAGTAATGAGTTTAGTTTTGATGGGGACAGGGGA 773

* *** ** *** ******* * *******************************

BnaA09.CRTISO AACTAAACTTGATAACTCAGGCGTTGAAGGCAGTTGGTCGTGAGATGGAGGTTATACCTG 888

BnaC08.CRTISO ATCTAAACTTGATAACTCAGGCGTTGAAGGCAGTTGGTCGTGAGATGGAAGTTATACCCG 833

* *********************************************** ******** *

BnaA09.CRTISO ATCCCACCACTGTCCATTTCCATCTTCCCAATGATCTCTCTGTTCAGGTTCATAGAGAGT 948

BnaC08.CRTISO ATCCCACCACTGTTCATTTCCATCTCCCCAGTGATCTCTCTGTTCAGGTTCATAGAGAGT 893

************* *********** **** *****************************

BnaA09.CRTISO ATGATGAGTTCGTTAATGAGCTTATTAGCAAGTTTCCGCACGAGAAGGAAGGGATTCTTG 1008

BnaC08.CRTISO ATGATGAGTTCGTTAATGAGCTTATTAGCAAGTTTCCTCACGAGAAGGAAGGGATTCTTG 953

************************************* **********************

BnaA09.CRTISO GATTCTATGGCGTCTGCTGGAAGGTTCTGATGTTACTTTTTACTTAATTGATTTTTTTTT 1068

BnaC08.CRTISO GATTCTATGGCGTCTGCTGGAAGGTTCTGATGTGACTTTTTTTACTTAATTG-------- 1005

********************************* *******

BnaA09.CRTISO TTTTGTACTGGTGTGTTGTGTTGTGCTGTGGGTTTATTATGTGTTACAACTTGGAACAGA 1128

BnaC08.CRTISO --------------AATATTTGTAAGAGTGTGTAATTTAAATATTACAAATTGGAACAGA 1051

* * * *** ** *** * ****** **********

BnaA09.CRTISO TCTTCAACTCATTGAACTCTTTGGAACTCAAGTCTCTTGAAGAACCTATCTACCTTTTTG 1188

BnaC08.CRTISO TCTTCAACTCATTGAACTCCCTGGAACTGAAGTCACTTGAAGAGCCTATCTACCTTTTTG 1111

******************* ******* ***** ******** ****************

BnaA09.CRTISO GACAGTTCTTTCAGAAGCCGCTTGAATGTTTGACACTCGGTTTGTCTTCTGAATAGTATC 1248

BnaC08.CRTISO GACAGTTCTTTCAGAAGCCGCTTGAATGTTTGACACTTGGTATTGGTTTTGATGTTTGT- 1170

************************************* *** * ** *** * *

BnaA09.CRTISO TAGACACTTGGATCATGTTTGATGTTTGTGTATTTATGTGAGTGTATGCTAACATTAATA 1308

BnaC08.CRTISO ---------------------------TTCTGTATCTATTAGTGTATACTGATATGAATC 1203

* * * * * * ******* ** * ** ***

BnaA09.CRTISO TTTTTATGTTGTTCTCTGTGATGTACAGCTTATTACTTGCCTCAAAATGCTGGGGACATA 1368

BnaC08.CRTISO TGTTCATGTTGTTCTGTTTGCTTTACAGCTTATTACTTGCCTCAAAATGCTGGGGATATA 1263

* ** ********** * ** * ********************************* ***

BnaA09.CRTISO GCTCGGAAGTACATAAAGGATCCTCAGTTACTGTCTTTCATTGACGCAGAGGTAAGAAAA 1428

BnaC08.CRTISO GCTAGGAAGTACATAAAGGATCCTCAGTTACTCTCTTTCATTGACGCAGAGGTGAGAAAA 1323

*** **************************** ******************** ******

BnaA09.CRTISO AATCA--TTTGGTCCAAACATGGACAGTTTATTTTACTCAGTTTTATAAAACAGACCTAT 1486

BnaC08.CRTISO AATGTCATTTGGTCCAAACATGCACAGTTTATTT------------TACACCATTTGTAT 1371

*** *************** *********** ** * ** ***

BnaA09.CRTISO TTTTTCCTGCAGTGTTTCATTGTGAGCACAGTGAATGCTTTGCAGACGCCAATGATCAAT 1546

BnaC08.CRTISO TTTTGCCTGCAGTGTTTCATTGTGAGCACAGTCAATGCTTTGCAGACGCCAATGATCAAT 1431

**** *************************** ***************************

BnaA09.CRTISO GCAAGTATGGTAGGATTCTTGTTTTTGGGGATTTTGGTGTCTACCCCATGTTTCCCTTTC 1606

BnaC08.CRTISO GCAAGTATGGTAGGATTCTTGTTCTGACATTTGGT---GTCTACC-CATGTTTCCC---- 1483

*********************** * * * ******* **********

BnaA09.CRTISO TGTTCAGTTTCTAAGTGTTCAAGGACAGGTTTTATGTGACAGGCACTATGGAGGGATTAA 1666

BnaC08.CRTISO TTTCTGTTTCTCATGTGTTCAAGGACAGGTTTTATGTGACAGGCACTATGGAGGAATTAA 1543

* * ** * **************************************** *****

BnaA09.CRTISO CTACCCTGTTGGTGGTGTTGGTGGGATTGCAAGGTCTTTAGCAGGAGGACTAGTTGATCA 1726

BnaC08.CRTISO CTACCCTGTTGGTGGTGTTGGTGGGATTGCAAAGTCTTTGGCAGATGGGCTTGTTGATCA 1603

******************************** ****** **** ** ** ********

BnaA09.CRTISO AGGAAGTGAAATACTCTACAAAGCTAATGTGAAAAGCATAATTCTTGATGATGGAAAGGC 1786

BnaC08.CRTISO AGGCAGTGAAATACTCTACAAAGCTAATGTCAAAAGCATAATACTTGATGATGGAAAGGC 1663

*** ************************** *********** *****************

BnaA09.CRTISO TGTAAGTTTCTGTATAACTCTTGCTACATTGATTCCAAGTTGTTGTTCTAAAAATCGATC 1846

BnaC08.CRTISO TGTAAGTTTCTGTATGACCCTTGCTACATATATTCCAAGTTTTTGTTCTTAAATTCCATC 1723

*************** ** ********** ********** ******* *** ** ***

BnaA09.CRTISO TAGACCTCCGACTAGTCAGCAAATCAGTCCTAAAGAAAACAATTTTTCGGTTTAGATGCT 1906

BnaC08.CRTISO TAGGCGTCCCCCTAGTCGGCAAATCGGACTTCAAGAAAACATTTTTTTTAGAATGATTTT 1783

*** * *** ****** ******* * * * ********* ***** *** *

BnaA09.CRTISO CAAAATA-TCGGTCTAGGCGCCCGCATAATCAATAATCCCATATAAAGCTTCCAACTACC 1965

BnaC08.CRTISO CTTAAAAATCGGTTTAGGCGCTCAAAGTA-------------------------TCGGTC 1818

* ** * ***** ******* * * * * *

BnaA09.CRTISO CCTACCGTTCTTGAACATTGATTCTTGCTTAACCTCTTGAAATTCTTATTTGGTTGT--- 2022

BnaC08.CRTISO TAGTGATTTCTTGAACATTGATTCTTGCTTAATTTTTTGAAATTCCTATTTGGTTGTGTG 1878

************************* * ********* ***********

BnaA09.CRTISO GTTTTCTATCTTCAGGTGGGTGTAAGGCTAGCAGATGGAAGAGAATTCTTCGCTAAAACG 2082

BnaC08.CRTISO TTTTTTTATCTTCAGGTTGGTGTAAGGCTAGCAGATGGAAGAGAATTCTTCGCTAAAACG 1938

**** *********** ******************************************

BnaA09.CRTISO ATAATTTCTAATGCTACAAGATGGGATACGTTTGGTAAGAGAAAACAATGACTTGCCTAA 2142

BnaC08.CRTISO ATAATCTCTAATGCTACAAGATGGGATACGTTTGGTAAGAGAAGACAATGACTTGCTTAA 1998

***** ************************************* ************ ***

BnaA09.CRTISO GTGTTGAATAGGTGTCTTGTTTGGTAAGAATACTTAGCATAGTATTACTAATGATGTAGG 2202

BnaC08.CRTISO G--TTGAATAGGTGTCTTGTTTTGTAAGAATACCTAGCACAGTATTACTAATGATGTAGG 2056

* ******************* ********** ***** ********************

BnaA09.CRTISO GAAGCTGTTGAAAGGAGAAAAGCTTCCAAAAGAAGAAGAAAACTTCCAGAAAGTCTACGT 2262

BnaC08.CRTISO GAAGCTGCTGAAAGGAGAAAAGCTTCCAAAGGAAGAAGAAAACTTCCAGAAAGTCTATGT 2116

******* ********************** ************************** **

BnaA09.CRTISO GAAGGCTCCATCGTTTCTTTCAATCCACATGGGTGTTAAAGCAGAGGTTCTCCCTCCAGA 2322

BnaC08.CRTISO GAAGGCTCCATCATTTCTCTCAATCCATATGGGTGTTAAAGCAGAGGTTCTCCCTCCAGA 2176

************ ***** ******** ********************************

BnaA09.CRTISO TACAGATTGCCATCATTTCGTACTTGAGGTTTGTTAGTTTCTTACTAAGATCTATTATTC 2382

BnaC08.CRTISO TACAGATTGCCATCATTTCGTACTTGAGGTTTGTTAGTTTCTTACTAAGATATATTATTG 2236

*************************************************** *******

BnaA09.CRTISO CCTTTGACAGATTCATAGTTATTTGAATAGTGGTTATGTTCTTTTGTTGCAACCTGTTAG 2442

BnaC08.CRTISO CCTTTGACAGAGTCATAGTTGTTTGAATGGTGGTTATGTTCTTTTGTTGCAACCTGTTAG 2296

*********** ******** ******* *******************************

BnaA09.CRTISO GATGATTGGAAGAATCTGGAGGAGCCTTATGGCAGTATCTTCCTCAGCATCCCAACCATT 2502

BnaC08.CRTISO GATGATTGGAAGAATTTGGAGGAGCCTTATGGCAGTATCTTCCTCAGCATTCCAACTATT 2356

*************** ********************************** ***** ***

BnaA09.CRTISO CTTGATCCATCCTTGGCTCCAGATGGTCGACATATACTCCACATATTTACAACTTCTTCC 2562

BnaC08.CRTISO CTTGATCCATATTTGGCTCCAGATGGTCGACATATACTGCACATATTTACAACCTCTTCC 2416

********** ************************** ************** ******

BnaA09.CRTISO ATTGAAGATTGGGAGGTAAGAGGCTGATCTTAAAAGAGTGAGACCAAGCATCAATTATAT 2622

BnaC08.CRTISO ATCGAAGATTGGGAGGTAAGGCTAATTTTATTGAAT-------------GAGTATGGATA 2463

** ***************** * * ** **

BnaA09.CRTISO ATGCCATCTTATTTGACCGTTTCATCTAAATTTTGGTACTATAGGGACTCACTCCAAAAG 2682

BnaC08.CRTISO TGCCCATCTTATTTGACCATTTCATCTAAATTTTGGTACTATAGGGACTCACTCCAAAAG 2523

*************** *****************************************

BnaA09.CRTISO AGTATGAGGCTAAAAAAGAAGAGGTGGCAGCTGGAATCATACAGAGGCTAGAGAAAAAAC 2742

BnaC08.CRTISO AGTATGAAGCGAAAAAAGAAGAGGTGGCAGCTGGAATCATACAGAGGCTAGAGAAAAAAC 2583

******* ** *************************************************

BnaA09.CRTISO TGTTTCCTGGTCTCAGTTCATCTATTACTTTTAAGGAGGTTAGGCTTAATCTATGTTCCT 2802

BnaC08.CRTISO TGTTTCCTGGGCTCAGTTCATCTATTACGTTTAAGGAGGTTTGGCTTAATCTTTGTTCCG 2643

********** ***************** ************ ********** ******

BnaA09.CRTISO GCCTA--------------------GATTTGCCATTCACATCTGTATATCTAAGGTGTTT 2842

BnaC08.CRTISO TTGCATGAGAGCAAGCCTAGGCCTTAATTTGAGTATTTAGCCTGTATATCTAAGGTGTTT 2703

* ***** * *******************

BnaA09.CRTISO TTAATAAGGCAGGTGGGCACACCAAGAACACACAGACGATATCTTGCTCGGGACAAGGGA 2902

BnaC08.CRTISO TTAATAAGGCAGGTGGGCACACCAAGAACACACAGGCGGTATCTTGCTCGGGACAAGGGA 2763

*********************************** ** *********************

BnaA09.CRTISO ACGTATGGACCAATGCCAAGAGGAACACCAAAAGGTTTACTGGGCATGCCGTTTAACACA 2962

BnaC08.CRTISO ACGTATGGACCAATGCCAAGAGGAACACCAAAAGGTTTACTAGGCATGCCGTTTAATACA 2823

***************************************** ************** ***

BnaA09.CRTISO ACTGTAAGTCAAAAGAAACGATTAGATGGTTCCCTTCTTGGGCTTAA------CTAAATG 3016

BnaC08.CRTISO ACTGTAAGTCAAAAGAAAAGAGTAGATGGTTCTGGGGCTTAACTAAATGATTTGGCTAAT 2883

****************** ** ********** * ** ** *

BnaA09.CRTISO GTTTTGGCTGATTGTAATAGGCTATAGATGGTTTGTACTGCGTTGGGGATAGTTGTTTTC 3076

BnaC08.CRTISO GGTTTGGCTGATTGTAATAGGCTATAGATGGTTTGTACTGCGTTGGGGATAGTTGTTTTC 2943

* **********************************************************

BnaA09.CRTISO CTGGTCAGGGAGTTATAGCTGTGGCTTTCTCAGGAGTGATGTGTGCTCATCGTGTAGCTG 3136

BnaC08.CRTISO CTGGTCAGGGAGTTATAGCTGTGGCTTTCTCAGGAGTGATGTGTGCTCATCGTGTAGCTG 3003

************************************************************

BnaA09.CRTISO CTGACATTGGTGAGAAAATTTGTTATAGACACTTGTTGATTGCTATGAGAGTAAGAAGCT 3196

BnaC08.CRTISO CTGACATTGGTGAGAAAATTTGTTATAGAGACTTGTTGATTGCTATGAGAGTAAGAAGCT 3063

***************************** ******************************

BnaA09.CRTISO AACATTTTT-TTTTCATATTTTGATTTGTAGGGCTTGAGAGAAAATCAAAGGTACTTGAT 3255

BnaC08.CRTISO AACATTTTTTTTTTCATATTTTGATTTGTAGGGCTTGAGAGAAAATCAAAGGTACTTGAT 3123

********* **************************************************

BnaA09.CRTISO GCTGGTCTTCTTGGTTTACTTGGTTGGTTAAGGACACTCGCGTAGTCTCTAAAGGGTACA 3315

BnaC08.CRTISO GCTGGTCTTCTTGGTTTACTTGGTTGGTTAAGGACACTCGCATAGTCTCTAAGGGTTACA 3183

***************************************** ********** ** ****

BnaA09.CRTISO GGACACAAAGCTAGTGGAAGTGTTGCTCAAGTCCATGAGAGCGGCTTTA-CTATGAACAA 3374

BnaC08.CRTISO GGACACAAAGCTAGTGGAAGTGTTGCTCAAGTCCATGAGAGCGGCTTTTACAATGAACAA 3243

************************************************ * ********

BnaA09.CRTISO CCTTTTTTAATTGTTGTTTAGTATCAAGAAGAGTTGCACATAGATAAGAAAAATATTTTT 3434

BnaC08.CRTISO CCCTTTTTATTTGTTGTTTAGGATCAAGAAGAGTTGCACATAGATAAGAAAAATATTTTT 3303

** ****** *********** **************************************

BnaA09.CRTISO ACCATATTCAATACTTTTTTGTCTTTTCTCTTCATACACTGATAAAATCAAATTAAGGAA 3494

BnaC08.CRTISO CCATTATTCAATACTTTTTTGTCTCTC-TCTTCATACACTGATAAAATCAAATAAAAGGA 3362

* ******************** * ************************* ** * *

BnaA09.CRTISO TCTAATCCT 3503

BnaC08.CRTISO ATCTAA--- 3368

**Fig. S3** Sequence alignment of two functional *BnaCRTISO* gene copies in J9707. The target sequences are underlined with the PAM highlighted in red.


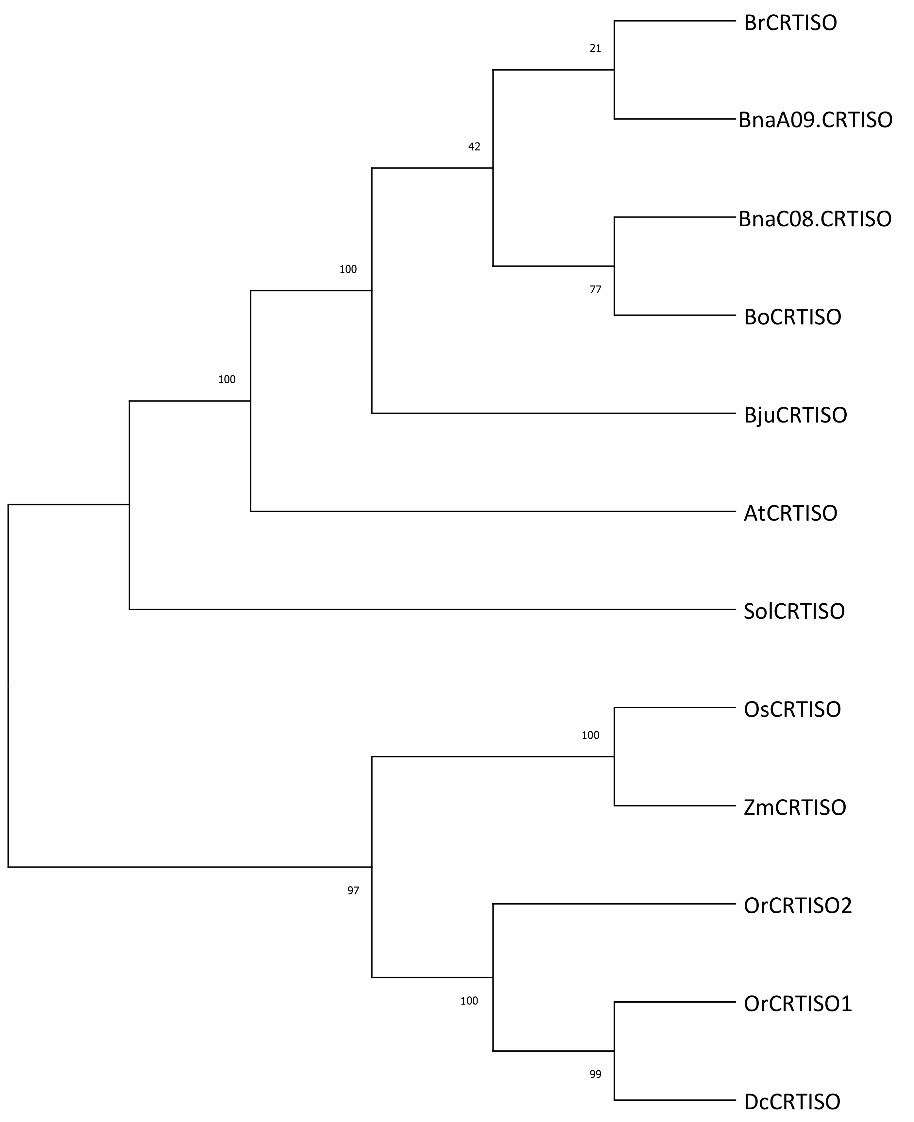


**Fig. S4 Phylogenetic tree showing the sequence relationship among *CRTISO* homologs identified from various plant species.** Protein sequences are from GenBank with the following accession numbers: AtCRTISO (Q9M9Y8) in *Arabidopsis thaliana*; BnaA09.CRTISO (XP_013716452.1) and BnaC08.CRTISO (CDY09954.1) in *Brassica napus*; BoCRTISO (A0A0D3DYP6) in *Brassica oleracea*; BjuCRTISO ([KC907718.1](https://www.ncbi.nlm.nih.gov/nucleotide/KC907718.1?report=genbank&log$=nucltop&blast_rank=1&RID=DBEE9ZKF016)) in *Brassica* *juncea*; BrCRTISO (VDC64404.1) in *Brassica rapa*; *SolCRTISO* (Q8S4R4) in *Solanum lycopersicum*; OncCRTISO1 (Q52QW3) and OncCRTISO2 (Q52QW2) in *Oncidium hybrid cultivar*; DcCRTISO (Q52QW3) in *Daucus carota*; OsCRTISO (Q2R2A6) in *Oryza sativa subsp. japonica*; ZmCRTISO (C1JFF5) in *Zea mays*.

A

B

**Fig. S5** Expression pattern of BnaCRTISO in rapeseed. (A): Relative gene expression of BnaCRTISO in various tissues of ZS11; (B): The expression level of CRTISO from qRT-PCR in the petal of J9707.

**A**

**CRTISO-94-15-5-2 aacc T3**

>aa

BnaA09.CRTISO MNLCLHNPVTCADRSSSLFSALKTSNNKLGTSKFGFLKNRKKNHVVAVRSVSSTAVEERT 60

aa MNLCLHNPVNVC* 12

BnaA09.CRTISO KRESGGGESKVYDAIVIGSGIGGLVAATQLAVKEAKVLVLEKYLIPGGSSGYYERDGYTF 120

BnaA09.CRTISO DVGSSVMFGFSDKGKLNLITQALKAVGREMEVIPDPTTVHFHLPNDLSVQVHREYDEFVN 180

BnaA09.CRTISO ELISKFPHEKEGILGFYGVCWKIFNSLNSLELKSLEEPIYLFGQFFQKPLECLTLAYYLP 240

BnaA09.CRTISO QNAGDIARKYIKDPQLLSFIDAECFIVSTVNALQTPMINASMVLCDRHYGGINYPVGGVG 300

BnaA09.CRTISO GIARSLAGGLVDQGSEILYKANVKSIILDDGKAVGVRLADGREFFAKTIISNATRWDTFG 360

BnaA09.CRTISO KLLKGEKLPKEEENFQKVYVKAPSFLSIHMGVKAEVLPPDTDCHHFVLEDDWKNLEEPYG 420

BnaA09.CRTISO SIFLSIPTILDPSLAPDGRHILHIFTTSSIEDWEGLTPKEYEAKKEEVAAGIIQRLEKKL 480

BnaA09.CRTISO FPGLSSSITFKEVGTPRTHRRYLARDKGTYGPMPRGTPKGLLGMPFNTTAIDGLYCVGDS 540

BnaA09.CRTISO CFPGQGVIAVAFSGVMCAHRVAADIGLERKSKVLDAGLLGLLGWLRTLA 589

>cc

BnaC08.CRTISO MNLCLRNPVTCADRSSSLFSALKTSNNKLGTSKFGFLKNPKKNHVVAVRSVSSTAVVEER 60

cc MNLCLRNPV--------------------------------------------------- 9

BnaC08.CRTISO TKRESGGGESKVYDAIVIGSGIGGLVAATQLAVKEAKVLVLEKYLIPGGSSGYYERDGYT 120

cc ---------SKVYDAIVIGSGIGGLVAATQLAVKEAKVLVLEKYLIPGGSSGYYERDGYT 60

BnaC08.CRTISO FDVGSSVMFGFSDKGNLNLITQALKAVGREMEVIPDPTTVHFHLPSDLSVQVHREYDEFV 180

cc FDVGSSVMFGFSDKGNLNLITQALKAVGREMEVIPDPTTVHFHLPSDLSVQVHREYDEFV 120

BnaC08.CRTISO NELISKFPHEKEGILGFYGVCWKIFNSLNSLELKSLEEPIYLFGQFFQKPLECLTLAYYL 240

cc NELISKFPHEKEGILGFYGVCWKIFNSLNSLELKSLEEPIYLFGQFFQKPLECLTLAYYL 180

BnaC08.CRTISO PQNAGDIARKYIKDPQLLSFIDAECFIVSTVNALQTPMINASMVLCDRHYGGINYPVGGV 300

cc PQNAGDIARKYIKDPQLLSFIDAECFIVSTVNALQTPMINASMVLCDRHYGGINYPVGGV 240

BnaC08.CRTISO GGIAKSLADGLVDQGSEILYKANVKSIILDDGKAVGVRLADGREFFAKTIISNATRWDTF 360

cc GGIAKSLADGLVDQGSEILYKANVKSIILDDGKAVGVRLADGREFFAKTIISNATRWDTF 300

BnaC08.CRTISO GKLLKGEKLPKEEENFQKVYVKAPSFLSIHMGVKAEVLPPDTDCHHFVLEDDWKNLEEPY 420

cc GKLLKGEKLPKEEENFQKVYVKAPSFLSIHMGVKAEVLPPDTDCHHFVLEDDWKNLEEPY 360

BnaC08.CRTISO GSIFLSIPTILDPYLAPDGRHILHIFTTSSIEDWEGLTPKEYEAKKEEVAAGIIQRLEKK 480

cc GSIFLSIPTILDPYLAPDGRHILHIFTTSSIEDWEGLTPKEYEAKKEEVAAGIIQRLEKK 420

BnaC08.CRTISO LFPGLSSSITFKEVGTPRTHRRYLARDKGTYGPMPRGTPKGLLGMPFNTTAIDGLYCVGD 540

cc LFPGLSSSITFKEVGTPRTHRRYLARDKGTYGPMPRGTPKGLLGMPFNTTAIDGLYCVGD 480

BnaC08.CRTISO SCFPGQGVIAVAFSGVMCAHRVAADIGLERKSKVLDAGLLGLLGWLRTLA* 590

cc SCFPGQGVIAVAFSGVMCAHRVAADIGLERKSKVLDAGLLGLLGWLRTLA* 530

**B**

**CRTISO-94-10-6-1 aacc T3**

>aa

BnaA09.CRTISO MNLCLHNPVTCADRSSSLFSALKTSNNKLGTSKFGFLKNRKKNHVVAVRSVSSTAVEERT 60

aa MNLCLHNPVTCADRSSSLFSALKTSNNKLGTSKFGFLKNRKKNHVVAVRSVSSTAVEERT 60

BnaA09.CRTISO KRESGGGESKVYDAIVIGSGIGGLVAATQLAVKEAKVLVLEKYLIPGGSSGYYERDGYTF 120

aa KRESRRRE* 68

BnaA09.CRTISO DVGSSVMFGFSDKGKLNLITQALKAVGREMEVIPDPTTVHFHLPNDLSVQVHREYDEFVN 180

BnaA09.CRTISO ELISKFPHEKEGILGFYGVCWKIFNSLNSLELKSLEEPIYLFGQFFQKPLECLTLAYYLP 240

BnaA09.CRTISO QNAGDIARKYIKDPQLLSFIDAECFIVSTVNALQTPMINASMVLCDRHYGGINYPVGGVG 300

BnaA09.CRTISO GIARSLAGGLVDQGSEILYKANVKSIILDDGKAVGVRLADGREFFAKTIISNATRWDTFG 360

BnaA09.CRTISO KLLKGEKLPKEEENFQKVYVKAPSFLSIHMGVKAEVLPPDTDCHHFVLEDDWKNLEEPYG 420

BnaA09.CRTISO SIFLSIPTILDPSLAPDGRHILHIFTTSSIEDWEGLTPKEYEAKKEEVAAGIIQRLEKKL 480

BnaA09.CRTISO FPGLSSSITFKEVGTPRTHRRYLARDKGTYGPMPRGTPKGLLGMPFNTTAIDGLYCVGDS 540

BnaA09.CRTISO CFPGQGVIAVAFSGVMCAHRVAADIGLERKSKVLDAGLLGLLGWLRTLA* 589

>cc

BnaC08.CRTISO MNLCLRNPVTCADRSSSLFSALKTSNNKLGTSKFGFLKNPKKNHVVAVRSVSSTAVVEER 60

cc MNLCLRNPVSKVYDAIVIGSGIGGLVAATQLAVKEAKVLVLEKYLIPGGSSGYYERDGYT 60

BnaC08.CRTISO TKRESGGGESKVYDAIVIGSGIGGLVAATQLAVKEAKVLVLEKYLIPGGSSGYYERDGYT 120

BnaC08.CRTISO FDVGSSVMFGFSDKGNLNLITQALKAVGREMEVIPDPTTVHFHLPSDLSVQVHREYDEFV 180

cc FDVGSSVMFGFSDKGNLNLITQALKAVGREMEVIPDPTTVHFHLPSDLSVQVHREYDEFV 120

BnaC08.CRTISO NELISKFPHEKEGILGFYGVCWKIFNSLNSLELKSLEEPIYLFGQFFQKPLECLTLAYYL 240

cc NELISKFPHEKEGILGFYGVCWKIFNSLNSLELKSLEEPIYLFGQFFQKPLECLTLAYYL 180

BnaC08.CRTISO PQNAGDIARKYIKDPQLLSFIDAECFIVSTVNALQTPMINASMVLCDRHYGGINYPVGGV 300

cc PQNAGDIARKYIKDPQLLSFIDAECFIVSTVNALQTPMINASMVLCDRHYGGINYPVGGV 240

BnaC08.CRTISO GGIAKSLADGLVDQGSEILYKANVKSIILDDGKAVGVRLADGREFFAKTIISNATRWDTF 360

cc GGIAKSLADGLVDQGSEILYKANVKSIILDDGKAVGVRLADGREFFAKTIISNATRWDTF 300

BnaC08.CRTISO GKLLKGEKLPKEEENFQKVYVKAPSFLSIHMGVKAEVLPPDTDCHHFVLEDDWKNLEEPY 420

cc GKLLKGEKLPKEEENFQKVYVKAPSFLSIHMGVKAEVLPPDTDCHHFVLEDDWKNLEEPY 360

BnaC08.CRTISO GSIFLSIPTILDPYLAPDGRHILHIFTTSSIEDWEGLTPKEYEAKKEEVAAGIIQRLEKK 480

cc GSIFLSIPTILDPYLAPDGRHILHIFTTSSIEDWEGLTPKEYEAKKEEVAAGIIQRLEKK 420

BnaC08.CRTISO LFPGLSSSITFKEVGTPRTHRRYLARDKGTYGPMPRGTPKGLLGMPFNTTAIDGLYCVGD 540

cc LFPGLSSSITFKEVGTPRTHRRYLARDKGTYGPMPRGTPKGLLGMPFNTTAIDGLYCVGD 480

BnaC08.CRTISO SCFPGQGVIAVAFSGVMCAHRVAADIGLERKSKVLDAGLLGLLGWLRTLA* 590

cc SCFPGQGVIAVAFSGVMCAHRVAADIGLERKSKVLDAGLLGLLGWLRTLA* 530

**C**

**CRTISO-48-8-13-1 aacc T3**

>aa

BnaA09.CRTISO MNLCLHNPVTCADRSSSLFSALKTSNNKLGTSKFGFLKNRKKNHVVAVRSVSSTAVEERT 60

aa MNLCLHNPVTCADRSSSLFSALKTSNNKLGTSKFGFLKNRKKNHVVAVRSVSSTAVEERT 60

BnaA09.CRTISO KRESGGGESKVYDAIVIGSGIGGLVAATQLAVKEAKVLVLEKYLIPGGSSGYYERDGYTF 120

aa KRESGEERVKCTTQSSSGLGLED* 83

BnaA09.CRTISO DVGSSVMFGFSDKGKLNLITQALKAVGREMEVIPDPTTVHFHLPNDLSVQVHREYDEFVN 180

BnaA09.CRTISO ELISKFPHEKEGILGFYGVCWKIFNSLNSLELKSLEEPIYLFGQFFQKPLECLTLAYYLP 240

BnaA09.CRTISO QNAGDIARKYIKDPQLLSFIDAECFIVSTVNALQTPMINASMVLCDRHYGGINYPVGGVG 300

BnaA09.CRTISO GIARSLAGGLVDQGSEILYKANVKSIILDDGKAVGVRLADGREFFAKTIISNATRWDTFG 360

BnaA09.CRTISO KLLKGEKLPKEEENFQKVYVKAPSFLSIHMGVKAEVLPPDTDCHHFVLEDDWKNLEEPYG 420

BnaA09.CRTISO SIFLSIPTILDPSLAPDGRHILHIFTTSSIEDWEGLTPKEYEAKKEEVAAGIIQRLEKKL 480

BnaA09.CRTISO FPGLSSSITFKEVGTPRTHRRYLARDKGTYGPMPRGTPKGLLGMPFNTTAIDGLYCVGDS 540

BnaA09.CRTISO CFPGQGVIAVAFSGVMCAHRVAADIGLERKSKVLDAGLLGLLGWLRTLA* 589

>cc

BnaC08.CRTISO MNLCLRNPVTCADRSSSLFSALKTSNNKLGTSKFGFLKNPKKNHVVAVRSVSSTAVVEER 60

cc MNLCLRNPVTCADRSSSLFSALKTSNNKLGTSKFGFLKNPKKNHVVAVRSVSSTAVVEER 60

BnaC08.CRTISO TKRESGGGESKVYDAIVIGSGIGGLVAATQLAVKEAKVLVLEKYLIPGGSSGYYERDGYT 120

cc TKRESEEERVKYTTQSSSGLG* 81

BnaC08.CRTISO FDVGSSVMFGFSDKGNLNLITQALKAVGREMEVIPDPTTVHFHLPSDLSVQVHREYDEFV 180

BnaC08.CRTISO NELISKFPHEKEGILGFYGVCWKIFNSLNSLELKSLEEPIYLFGQFFQKPLECLTLAYYL 240

BnaC08.CRTISO PQNAGDIARKYIKDPQLLSFIDAECFIVSTVNALQTPMINASMVLCDRHYGGINYPVGGV 300

BnaC08.CRTISO GGIAKSLADGLVDQGSEILYKANVKSIILDDGKAVGVRLADGREFFAKTIISNATRWDTF 360

BnaC08.CRTISO GKLLKGEKLPKEEENFQKVYVKAPSFLSIHMGVKAEVLPPDTDCHHFVLEDDWKNLEEPY 420

BnaC08.CRTISO GSIFLSIPTILDPYLAPDGRHILHIFTTSSIEDWEGLTPKEYEAKKEEVAAGIIQRLEKK 480

BnaC08.CRTISO LFPGLSSSITFKEVGTPRTHRRYLARDKGTYGPMPRGTPKGLLGMPFNTTAIDGLYCVGD 540

BnaC08.CRTISO SCFPGQGVIAVAFSGVMCAHRVAADIGLERKSKVLDAGLLGLLGWLRTLA* 590

**D**

**CRTISO-7-2-2-4 aacc T3**

>aa

BnaA09.CRTISO MNLCLHNPVTCADRSSSLFSALKTSNNKLGTSKFGFLKNRKKNHVVAVRSVSSTAVEERT 60

aa MNLCLHNPVTCADRSSSLFSALKTSNNKLGTSKFGFLKNRKKNHVVAVRSVSSTAVEERT 60

BnaA09.CRTISO KRESGGGESKVYDAIVIGSGIGGLVAATQLAVKEAKVLVLEKYLIPGGSSGYYERDGYTF 120

aa KRVKCTTQSSSGLGLED* 77

BnaA09.CRTISO DVGSSVMFGFSDKGKLNLITQALKAVGREMEVIPDPTTVHFHLPNDLSVQVHREYDEFVN 180

BnaA09.CRTISO ELISKFPHEKEGILGFYGVCWKIFNSLNSLELKSLEEPIYLFGQFFQKPLECLTLAYYLP 240

BnaA09.CRTISO QNAGDIARKYIKDPQLLSFIDAECFIVSTVNALQTPMINASMVLCDRHYGGINYPVGGVG 300

BnaA09.CRTISO GIARSLAGGLVDQGSEILYKANVKSIILDDGKAVGVRLADGREFFAKTIISNATRWDTFG 360

BnaA09.CRTISO KLLKGEKLPKEEENFQKVYVKAPSFLSIHMGVKAEVLPPDTDCHHFVLEDDWKNLEEPYG 420

BnaA09.CRTISO SIFLSIPTILDPSLAPDGRHILHIFTTSSIEDWEGLTPKEYEAKKEEVAAGIIQRLEKKL 480

BnaA09.CRTISO FPGLSSSITFKEVGTPRTHRRYLARDKGTYGPMPRGTPKGLLGMPFNTTAIDGLYCVGDS 540

BnaA09.CRTISO CFPGQGVIAVAFSGVMCAHRVAADIGLERKSKVLDAGLLGLLGWLRTLA* 589

>cc

BnaC08.CRTISO MNLCLRNPVTCADRSSSLFSALKTSNNKLGTSKFGFLKNPKKNHVVAVRSVSSTAVVEER 60

cc MNLCLRNPVTCADRSSSLFSALKTSNNKLGTSKFGFLKNPKKNHVVAVRSVSSTAVVEER 60

BnaC08.CRTISO TKRESGGGESKVYDAIVIGSGIGGLVAATQLAVKEAKVLVLEKYLIPGGSSGYYERDGYT 120

cc TKRERRRE* 68

BnaC08.CRTISO FDVGSSVMFGFSDKGNLNLITQALKAVGREMEVIPDPTTVHFHLPSDLSVQVHREYDEFV 180

BnaC08.CRTISO NELISKFPHEKEGILGFYGVCWKIFNSLNSLELKSLEEPIYLFGQFFQKPLECLTLAYYL 240

BnaC08.CRTISO PQNAGDIARKYIKDPQLLSFIDAECFIVSTVNALQTPMINASMVLCDRHYGGINYPVGGV 300

BnaC08.CRTISO GGIAKSLADGLVDQGSEILYKANVKSIILDDGKAVGVRLADGREFFAKTIISNATRWDTF 360

BnaC08.CRTISO GKLLKGEKLPKEEENFQKVYVKAPSFLSIHMGVKAEVLPPDTDCHHFVLEDDWKNLEEPY 420

BnaC08.CRTISO GSIFLSIPTILDPYLAPDGRHILHIFTTSSIEDWEGLTPKEYEAKKEEVAAGIIQRLEKK 480

BnaC08.CRTISO LFPGLSSSITFKEVGTPRTHRRYLARDKGTYGPMPRGTPKGLLGMPFNTTAIDGLYCVGD 540

BnaC08.CRTISO SCFPGQGVIAVAFSGVMCAHRVAADIGLERKSKVLDAGLLGLLGWLRTLA* 590

**E**

**CRTISO-48-10-4-1 aacc T3**

>aa

BnaA09.CRTISO MNLCLHNPVTCADRSSSLFSALKTSNNKLGTSKFGFLKNRKKNHVVAVRSVSSTAVEERT 60

aa MNLCLHNPVKEERVKCTTQSSSGLGLED* 28

BnaA09.CRTISO KRESGGGESKVYDAIVIGSGIGGLVAATQLAVKEAKVLVLEKYLIPGGSSGYYERDGYTF 120

BnaA09.CRTISO DVGSSVMFGFSDKGKLNLITQALKAVGREMEVIPDPTTVHFHLPNDLSVQVHREYDEFVN 180

BnaA09.CRTISO ELISKFPHEKEGILGFYGVCWKIFNSLNSLELKSLEEPIYLFGQFFQKPLECLTLAYYLP 240

BnaA09.CRTISO QNAGDIARKYIKDPQLLSFIDAECFIVSTVNALQTPMINASMVLCDRHYGGINYPVGGVG 300

BnaA09.CRTISO GIARSLAGGLVDQGSEILYKANVKSIILDDGKAVGVRLADGREFFAKTIISNATRWDTFG 360

BnaA09.CRTISO KLLKGEKLPKEEENFQKVYVKAPSFLSIHMGVKAEVLPPDTDCHHFVLEDDWKNLEEPYG 420

BnaA09.CRTISO SIFLSIPTILDPSLAPDGRHILHIFTTSSIEDWEGLTPKEYEAKKEEVAAGIIQRLEKKL 480

BnaA09.CRTISO FPGLSSSITFKEVGTPRTHRRYLARDKGTYGPMPRGTPKGLLGMPFNTTAIDGLYCVGDS 540

BnaA09.CRTISO CFPGQGVIAVAFSGVMCAHRVAADIGLERKSKVLDAGLLGLLGWLRTLA* 589

>cc

BnaC08.CRTISO MNLCLRNPVTCADRSSSLFSALKTSNNKLGTSKFGFLKNPKKNHVVAVRSVSSTAVVEER 60

cc MNLCLRNPVTCADRSSSLFSALKTSNNKLGTSKFGFLKNPKKNHVVAVRSVSSTAVVEER 60

BnaC08.CRTISO TKRESGGGESKVYDAIVIGSGIGGLVAATQLAVKEAKVLVLEKYLIPGGSSGYYERDGYT 120

cc TKRESEEERVKYTTQSSSGLG* 81

BnaC08.CRTISO FDVGSSVMFGFSDKGNLNLITQALKAVGREMEVIPDPTTVHFHLPSDLSVQVHREYDEFV 180

BnaC08.CRTISO NELISKFPHEKEGILGFYGVCWKIFNSLNSLELKSLEEPIYLFGQFFQKPLECLTLAYYL 240

BnaC08.CRTISO PQNAGDIARKYIKDPQLLSFIDAECFIVSTVNALQTPMINASMVLCDRHYGGINYPVGGV 300

BnaC08.CRTISO GGIAKSLADGLVDQGSEILYKANVKSIILDDGKAVGVRLADGREFFAKTIISNATRWDTF 360

BnaC08.CRTISO GKLLKGEKLPKEEENFQKVYVKAPSFLSIHMGVKAEVLPPDTDCHHFVLEDDWKNLEEPY 420

BnaC08.CRTISO GSIFLSIPTILDPYLAPDGRHILHIFTTSSIEDWEGLTPKEYEAKKEEVAAGIIQRLEKK 480

BnaC08.CRTISO LFPGLSSSITFKEVGTPRTHRRYLARDKGTYGPMPRGTPKGLLGMPFNTTAIDGLYCVGD 540

BnaC08.CRTISO SCFPGQGVIAVAFSGVMCAHRVAADIGLERKSKVLDAGLLGLLGWLRTLA* 590

**F**

**CRTISO-48-8-20-8 aacc T3**

>aa

BnaA09.CRTISO MNLCLHNPVTCADRSSSLFSALKTSNNKLGTSKFGFLKNRKKNHVVAVRSVSSTAVEERT 60

aa MNLCLHNPVTCADRSSSLFSALKTSNNKLGTSKFGFLKNRKKNHVVAVRSVSSTAVEERT 60

BnaA09.CRTISO KRESGGGESKVYDAIVIGSGIGGLVAATQLAVKEAKVLVLEKYLIPGGSSGYYERDGYTF 120

aa KRESGEERVKCTTQSSSGLGLED* 83

BnaA09.CRTISO DVGSSVMFGFSDKGKLNLITQALKAVGREMEVIPDPTTVHFHLPNDLSVQVHREYDEFVN 180

BnaA09.CRTISO ELISKFPHEKEGILGFYGVCWKIFNSLNSLELKSLEEPIYLFGQFFQKPLECLTLAYYLP 240

BnaA09.CRTISO QNAGDIARKYIKDPQLLSFIDAECFIVSTVNALQTPMINASMVLCDRHYGGINYPVGGVG 300

BnaA09.CRTISO GIARSLAGGLVDQGSEILYKANVKSIILDDGKAVGVRLADGREFFAKTIISNATRWDTFG 360

BnaA09.CRTISO KLLKGEKLPKEEENFQKVYVKAPSFLSIHMGVKAEVLPPDTDCHHFVLEDDWKNLEEPYG 420

BnaA09.CRTISO SIFLSIPTILDPSLAPDGRHILHIFTTSSIEDWEGLTPKEYEAKKEEVAAGIIQRLEKKL 480

BnaA09.CRTISO FPGLSSSITFKEVGTPRTHRRYLARDKGTYGPMPRGTPKGLLGMPFNTTAIDGLYCVGDS 540

BnaA09.CRTISO CFPGQGVIAVAFSGVMCAHRVAADIGLERKSKVLDAGLLGLLGWLRTLA* 589

>cc

BnaC08.CRTISO MNLCLRNPVTCADRSSSLFSALKTSNNKLGTSKFGFLKNPKKNHVVAVRSVSSTAVVEER 60

cc MNLCLRNPVTCADRSSSLFSALKTSNNKLGTSKFGFLKNPKKNHVVAVRSVSSTAVVEER 60

BnaC08.CRTISO TKRESGGGESKVYDAIVIGSGIGGLVAATQLAVKEAKVLVLEKYLIPGGSSGYYERDGYT 120

cc TKRESGRRRE* 70

BnaC08.CRTISO FDVGSSVMFGFSDKGNLNLITQALKAVGREMEVIPDPTTVHFHLPSDLSVQVHREYDEFV 180

BnaC08.CRTISO NELISKFPHEKEGILGFYGVCWKIFNSLNSLELKSLEEPIYLFGQFFQKPLECLTLAYYL 240

BnaC08.CRTISO PQNAGDIARKYIKDPQLLSFIDAECFIVSTVNALQTPMINASMVLCDRHYGGINYPVGGV 300

BnaC08.CRTISO GGIAKSLADGLVDQGSEILYKANVKSIILDDGKAVGVRLADGREFFAKTIISNATRWDTF 360

BnaC08.CRTISO GKLLKGEKLPKEEENFQKVYVKAPSFLSIHMGVKAEVLPPDTDCHHFVLEDDWKNLEEPY 420

BnaC08.CRTISO GSIFLSIPTILDPYLAPDGRHILHIFTTSSIEDWEGLTPKEYEAKKEEVAAGIIQRLEKK 480

BnaC08.CRTISO LFPGLSSSITFKEVGTPRTHRRYLARDKGTYGPMPRGTPKGLLGMPFNTTAIDGLYCVGD 540

BnaC08.CRTISO SCFPGQGVIAVAFSGVMCAHRVAADIGLERKSKVLDAGLLGLLGWLRTLA* 590

**G**

**CRTISO-48-8-20-6 aaCC T3**

>aa

BnaA09.CRTISO MNLCLHNPVTCADRSSSLFSALKTSNNKLGTSKFGFLKNRKKNHVVAVRSVSSTAVEERT 60

aa MNLCLHNPVTCADRSSSLFSALKTSNNKLGTSKFGFLKNRKKNHVVAVRSVSSTAVEERT 60

BnaA09.CRTISO KRESGGGESKVYDAIVIGSGIGGLVAATQLAVKEAKVLVLEKYLIPGGSSGYYERDGYTF 120

aa KRESGEERVKCTTQSSSGLGLED* 83

BnaA09.CRTISO DVGSSVMFGFSDKGKLNLITQALKAVGREMEVIPDPTTVHFHLPNDLSVQVHREYDEFVN 180

BnaA09.CRTISO ELISKFPHEKEGILGFYGVCWKIFNSLNSLELKSLEEPIYLFGQFFQKPLECLTLAYYLP 240

BnaA09.CRTISO QNAGDIARKYIKDPQLLSFIDAECFIVSTVNALQTPMINASMVLCDRHYGGINYPVGGVG 300

BnaA09.CRTISO GIARSLAGGLVDQGSEILYKANVKSIILDDGKAVGVRLADGREFFAKTIISNATRWDTFG 360

BnaA09.CRTISO KLLKGEKLPKEEENFQKVYVKAPSFLSIHMGVKAEVLPPDTDCHHFVLEDDWKNLEEPYG 420

BnaA09.CRTISO SIFLSIPTILDPSLAPDGRHILHIFTTSSIEDWEGLTPKEYEAKKEEVAAGIIQRLEKKL 480

BnaA09.CRTISO FPGLSSSITFKEVGTPRTHRRYLARDKGTYGPMPRGTPKGLLGMPFNTTAIDGLYCVGDS 540

BnaA09.CRTISO CFPGQGVIAVAFSGVMCAHRVAADIGLERKSKVLDAGLLGLLGWLRTLA* 589

>CC

BnaC08.CRTISO MNLCLRNPVTCADRSSSLFSALKTSNNKLGTSKFGFLKNPKKNHVVAVRSVSSTAVVEER 60

CC MNLCLRNPVTCADRSSSLFSALKTSNNKLGTSKFGFLKNPKKNHVVAVRSVSSTAVVEER 60

BnaC08.CRTISO TKRESGGGESKVYDAIVIGSGIGGLVAATQLAVKEAKVLVLEKYLIPGGSSGYYERDGYT 120

CC TKRE---RESKVYDAIVIGSGIGGLVAATQLAVKEAKVLVLEKYLIPGGSSGYYERDGYT 117

BnaC08.CRTISO FDVGSSVMFGFSDKGNLNLITQALKAVGREMEVIPDPTTVHFHLPSDLSVQVHREYDEFV 180

CC FDVGSSVMFGFSDKGNLNLITQALKAVGREMEVIPDPTTVHFHLPSDLSVQVHREYDEFV 177

BnaC08.CRTISO NELISKFPHEKEGILGFYGVCWKIFNSLNSLELKSLEEPIYLFGQFFQKPLECLTLAYYL 240

CC NELISKFPHEKEGILGFYGVCWKIFNSLNSLELKSLEEPIYLFGQFFQKPLECLTLAYYL 237

BnaC08.CRTISO PQNAGDIARKYIKDPQLLSFIDAECFIVSTVNALQTPMINASMVLCDRHYGGINYPVGGV 300

CC PQNAGDIARKYIKDPQLLSFIDAECFIVSTVNALQTPMINASMVLCDRHYGGINYPVGGV 297

BnaC08.CRTISO GGIAKSLADGLVDQGSEILYKANVKSIILDDGKAVGVRLADGREFFAKTIISNATRWDTF 360

CC GGIAKSLADGLVDQGSEILYKANVKSIILDDGKAVGVRLADGREFFAKTIISNATRWDTF 357

BnaC08.CRTISO GKLLKGEKLPKEEENFQKVYVKAPSFLSIHMGVKAEVLPPDTDCHHFVLEDDWKNLEEPY 420

CC GKLLKGEKLPKEEENFQKVYVKAPSFLSIHMGVKAEVLPPDTDCHHFVLEDDWKNLEEPY 417

BnaC08.CRTISO GSIFLSIPTILDPYLAPDGRHILHIFTTSSIEDWEGLTPKEYEAKKEEVAAGIIQRLEKK 480

CC GSIFLSIPTILDPYLAPDGRHILHIFTTSSIEDWEGLTPKEYEAKKEEVAAGIIQRLEKK 477

BnaC08.CRTISO LFPGLSSSITFKEVGTPRTHRRYLARDKGTYGPMPRGTPKGLLGMPFNTTAIDGLYCVGD 540

CC LFPGLSSSITFKEVGTPRTHRRYLARDKGTYGPMPRGTPKGLLGMPFNTTAIDGLYCVGD 537

BnaC08.CRTISO SCFPGQGVIAVAFSGVMCAHRVAADIGLERKSKVLDAGLLGLLGWLRTLA* 590

CC SCFPGQGVIAVAFSGVMCAHRVAADIGLERKSKVLDAGLLGLLGWLRTLA* 587

**H**

**CRTISO-48-8-21-3 aaCC T3**

>aa

BnaA09.CRTISO MNLCLHNPVTCADRSSSLFSALKTSNNKLGTSKFGFLKNRKKNHVVAVRSVSSTAVEERT 60

aa MNLCLHNPVTCADRSSSLFSALKTSNNKLGTSKFGFLKNRKKNHVVAVRSVSSTAVEERT 60

BnaA09.CRTISO KRESGGGESKVYDAIVIGSGIGGLVAATQLAVKEAKVLVLEKYLIPGGSSGYYERDGYTF 120

aa KRESGEERVKCTTQSSSGLGLED* 83

BnaA09.CRTISO DVGSSVMFGFSDKGKLNLITQALKAVGREMEVIPDPTTVHFHLPNDLSVQVHREYDEFVN 180

BnaA09.CRTISO ELISKFPHEKEGILGFYGVCWKIFNSLNSLELKSLEEPIYLFGQFFQKPLECLTLAYYLP 240

BnaA09.CRTISO QNAGDIARKYIKDPQLLSFIDAECFIVSTVNALQTPMINASMVLCDRHYGGINYPVGGVG 300

BnaA09.CRTISO GIARSLAGGLVDQGSEILYKANVKSIILDDGKAVGVRLADGREFFAKTIISNATRWDTFG 360

BnaA09.CRTISO KLLKGEKLPKEEENFQKVYVKAPSFLSIHMGVKAEVLPPDTDCHHFVLEDDWKNLEEPYG 420

BnaA09.CRTISO SIFLSIPTILDPSLAPDGRHILHIFTTSSIEDWEGLTPKEYEAKKEEVAAGIIQRLEKKL 480

BnaA09.CRTISO FPGLSSSITFKEVGTPRTHRRYLARDKGTYGPMPRGTPKGLLGMPFNTTAIDGLYCVGDS 540

BnaA09.CRTISO CFPGQGVIAVAFSGVMCAHRVAADIGLERKSKVLDAGLLGLLGWLRTLA* 589

**I**

**CRTISO-7-6-1-1 AAcc T3**

>AA

BnaA09.CRTISO MNLCLHNPVTCADRSSSLFSALKTSNNKLGTSKFGFLKNRKKNHVVAVRSVSSTAVEERT 60

AA MNLCLHNPVTCADRSSSLFSALKTSNNKLGTSKFGFLKNRKKNHVVAVRSVSSTAVEERT 60

BnaA09.CRTISO KRESGGGESKVYDAIVIGSGIGGLVAATQLAVKEAKVLVLEKYLIPGGSSGYYERDGYTF 120

AA KRESG--ESKVYDAIVIGSGIGGLVAATQLAVKEAKVLVLEKYLIPGGSSGYYERDGYTF 118

BnaA09.CRTISO DVGSSVMFGFSDKGKLNLITQALKAVGREMEVIPDPTTVHFHLPNDLSVQVHREYDEFVN 180

AA DVGSSVMFGFSDKGKLNLITQALKAVGREMEVIPDPTTVHFHLPNDLSVQVHREYDEFVN 178

BnaA09.CRTISO ELISKFPHEKEGILGFYGVCWKIFNSLNSLELKSLEEPIYLFGQFFQKPLECLTLAYYLP 240

AA ELISKFPHEKEGILGFYGVCWKIFNSLNSLELKSLEEPIYLFGQFFQKPLECLTLAYYLP 238

BnaA09.CRTISO QNAGDIARKYIKDPQLLSFIDAECFIVSTVNALQTPMINASMVLCDRHYGGINYPVGGVG 300

AA QNAGDIARKYIKDPQLLSFIDAECFIVSTVNALQTPMINASMVLCDRHYGGINYPVGGVG 298

BnaA09.CRTISO GIARSLAGGLVDQGSEILYKANVKSIILDDGKAVGVRLADGREFFAKTIISNATRWDTFG 360

AA GIARSLAGGLVDQGSEILYKANVKSIILDDGKAVGVRLADGREFFAKTIISNATRWDTFG 358

BnaA09.CRTISO KLLKGEKLPKEEENFQKVYVKAPSFLSIHMGVKAEVLPPDTDCHHFVLEDDWKNLEEPYG 420

AA KLLKGEKLPKEEENFQKVYVKAPSFLSIHMGVKAEVLPPDTDCHHFVLEDDWKNLEEPYG 418

BnaA09.CRTISO SIFLSIPTILDPSLAPDGRHILHIFTTSSIEDWEGLTPKEYEAKKEEVAAGIIQRLEKKL 480

AA SIFLSIPTILDPSLAPDGRHILHIFTTSSIEDWEGLTPKEYEAKKEEVAAGIIQRLEKKL 478

BnaA09.CRTISO FPGLSSSITFKEVGTPRTHRRYLARDKGTYGPMPRGTPKGLLGMPFNTTAIDGLYCVGDS 540

AA FPGLSSSITFKEVGTPRTHRRYLARDKGTYGPMPRGTPKGLLGMPFNTTAIDGLYCVGDS 538

BnaA09.CRTISO CFPGQGVIAVAFSGVMCAHRVAADIGLERKSKVLDAGLLGLLGWLRTLA* 589

AA CFPGQGVIAVAFSGVMCAHRVAADIGLERKSKVLDAGLLGLLGWLRTLA* 587

>cc

BnaC08.CRTISO MNLCLRNPVTCADRSSSLFSALKTSNNKLGTSKFGFLKNPKKNHVVAVRSVSSTAVVEER 60

cc MNLCLRNPVTCADRSSSLFSALKTSNNKLGTSKFGFLKNPKKNHVVAVRSVSSTAVVEER 60

BnaC08.CRTISO TKRESGGGESKVYDAIVIGSGIGGLVAATQLAVKEAKVLVLEKYLIPGGSSGYYERDGYT 120

cc TKRESGRRRE* 70

BnaC08.CRTISO FDVGSSVMFGFSDKGNLNLITQALKAVGREMEVIPDPTTVHFHLPSDLSVQVHREYDEFV 180

BnaC08.CRTISO NELISKFPHEKEGILGFYGVCWKIFNSLNSLELKSLEEPIYLFGQFFQKPLECLTLAYYL 240

BnaC08.CRTISO PQNAGDIARKYIKDPQLLSFIDAECFIVSTVNALQTPMINASMVLCDRHYGGINYPVGGV 300

BnaC08.CRTISO GGIAKSLADGLVDQGSEILYKANVKSIILDDGKAVGVRLADGREFFAKTIISNATRWDTF 360

BnaC08.CRTISO GKLLKGEKLPKEEENFQKVYVKAPSFLSIHMGVKAEVLPPDTDCHHFVLEDDWKNLEEPY 420

BnaC08.CRTISO GSIFLSIPTILDPYLAPDGRHILHIFTTSSIEDWEGLTPKEYEAKKEEVAAGIIQRLEKK 480

BnaC08.CRTISO LFPGLSSSITFKEVGTPRTHRRYLARDKGTYGPMPRGTPKGLLGMPFNTTAIDGLYCVGD 540

BnaC08.CRTISO SCFPGQGVIAVAFSGVMCAHRVAADIGLERKSKVLDAGLLGLLGWLRTLA* 590

**J**

**CRTISO-48-8-20-1 AAcc T3**

>cc

BnaC08.CRTISO MNLCLRNPVTCADRSSSLFSALKTSNNKLGTSKFGFLKNPKKNHVVAVRSVSSTAVVEER 60

cc MNLCLRNPVTCADRSSSLFSALKTSNNKLGTSKFGFLKNPKKNHVVAVRSVSSTAVVEER 60

BnaC08.CRTISO TKRESGGGESKVYDAIVIGSGIGGLVAATQLAVKEAKVLVLEKYLIPGGSSGYYERDGYT 120

cc TKRESGRRRE* 70

BnaC08.CRTISO FDVGSSVMFGFSDKGNLNLITQALKAVGREMEVIPDPTTVHFHLPSDLSVQVHREYDEFV 180

BnaC08.CRTISO NELISKFPHEKEGILGFYGVCWKIFNSLNSLELKSLEEPIYLFGQFFQKPLECLTLAYYL 240

BnaC08.CRTISO PQNAGDIARKYIKDPQLLSFIDAECFIVSTVNALQTPMINASMVLCDRHYGGINYPVGGV 300

BnaC08.CRTISO GGIAKSLADGLVDQGSEILYKANVKSIILDDGKAVGVRLADGREFFAKTIISNATRWDTF 360

BnaC08.CRTISO GKLLKGEKLPKEEENFQKVYVKAPSFLSIHMGVKAEVLPPDTDCHHFVLEDDWKNLEEPY 420

BnaC08.CRTISO GSIFLSIPTILDPYLAPDGRHILHIFTTSSIEDWEGLTPKEYEAKKEEVAAGIIQRLEKK 480

BnaC08.CRTISO LFPGLSSSITFKEVGTPRTHRRYLARDKGTYGPMPRGTPKGLLGMPFNTTAIDGLYCVGD 540

BnaC08.CRTISO SCFPGQGVIAVAFSGVMCAHRVAADIGLERKSKVLDAGLLGLLGWLRTLA* 590

**Fig. S6** The predicted amino acid sequences of *BnaCRTISO* homozygous mutants in T2 generation. CRTISO-94-15-5-2 (A), CRTISO-94-10-6-1 (B), CRTISO-48-8-13-1 (C), CRTISO-7-2-2-4 (D), CRTISO-48-10-4-1 (E), CRTISO-48-8-20-8 (F), are double-homozygous mutant lines of BnaCRTISO; CRTISO-48-8-20-6 (G), CRTISO-48-8-21-3 (H), are single-homozygous mutant lines of *BnaA09. CRTISO*; CRTISO-7-6-1-1 (I), CRTISO-48-8-20-1 (J), are single-homozygous mutant lines of *BnaC08. CRTISO*. Stars indicate stop codon, and numbers indicate amino acid positions; red letters indicate frame-shift amino acids; “-” indicate deletion of amino acid; “aa” and “cc” represent the homozygous mutated alleles of the target gene on *BnaA09. CRTISO* and *BnaC08. CRTISO*, respectively; “*aaCC*”, “*AAcc*” and “*aacc*” represent homozygous mutations of the target gene in *BnaA09. CRTISO*, *BnaC08. CRTISO* and both copies, respectively.


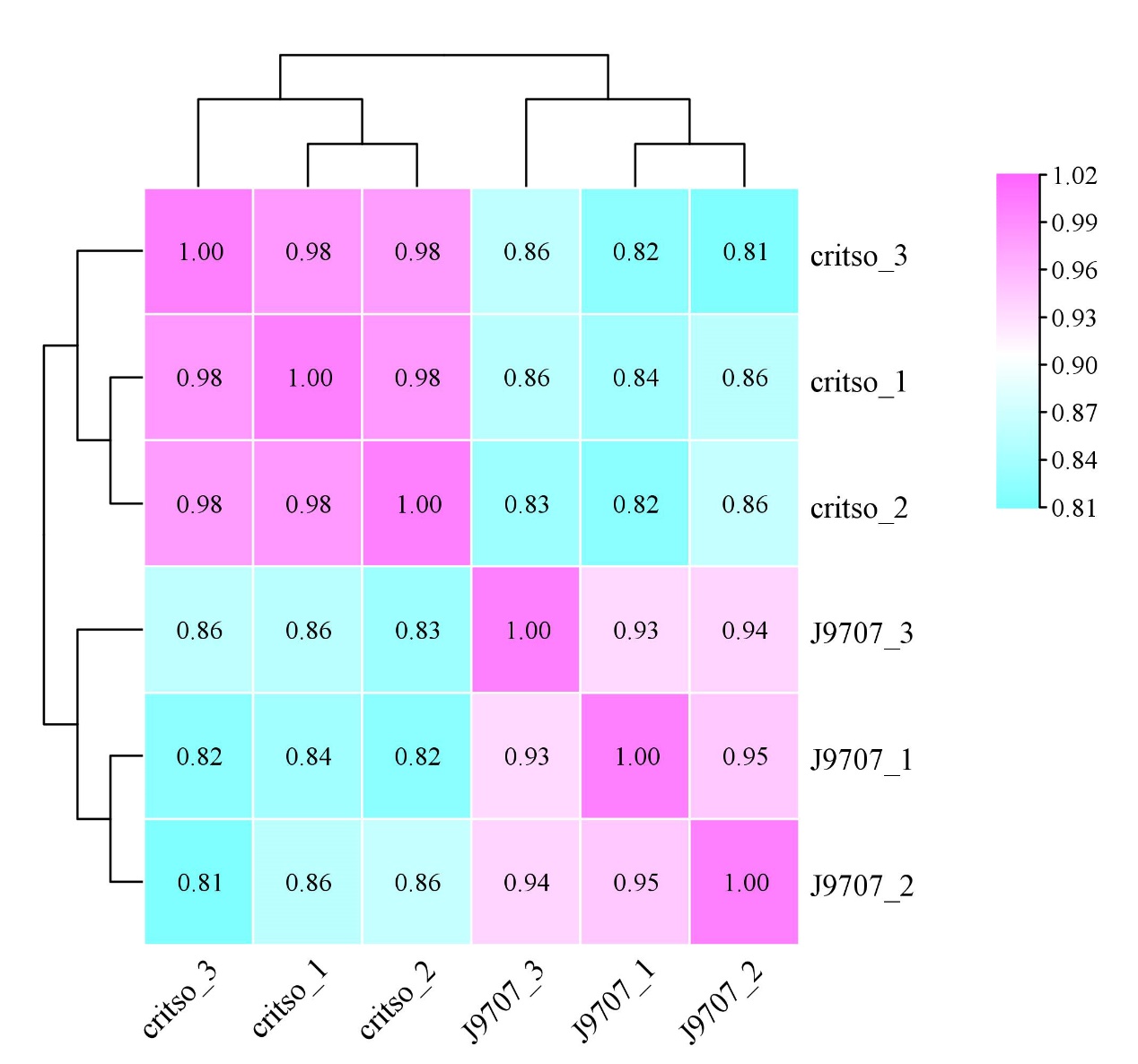


**Fig. S7** Pearson correlation coefficient among counts of transcriptome data.

**Fig. S8** Number of up-and down-DEGs between *BnaCRTISO* mutant (*crtiso*) and WT identified in developing flower.

A


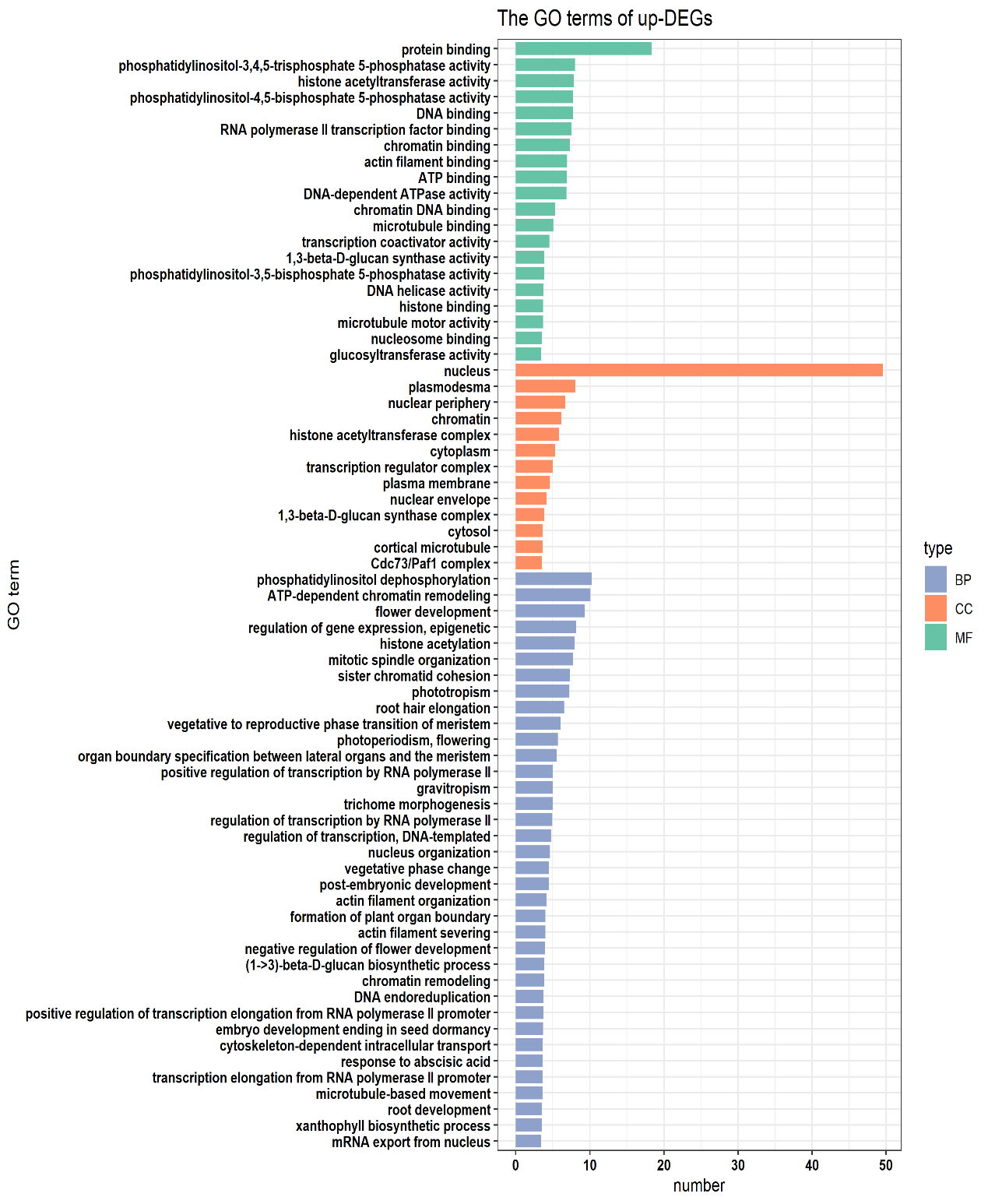


B


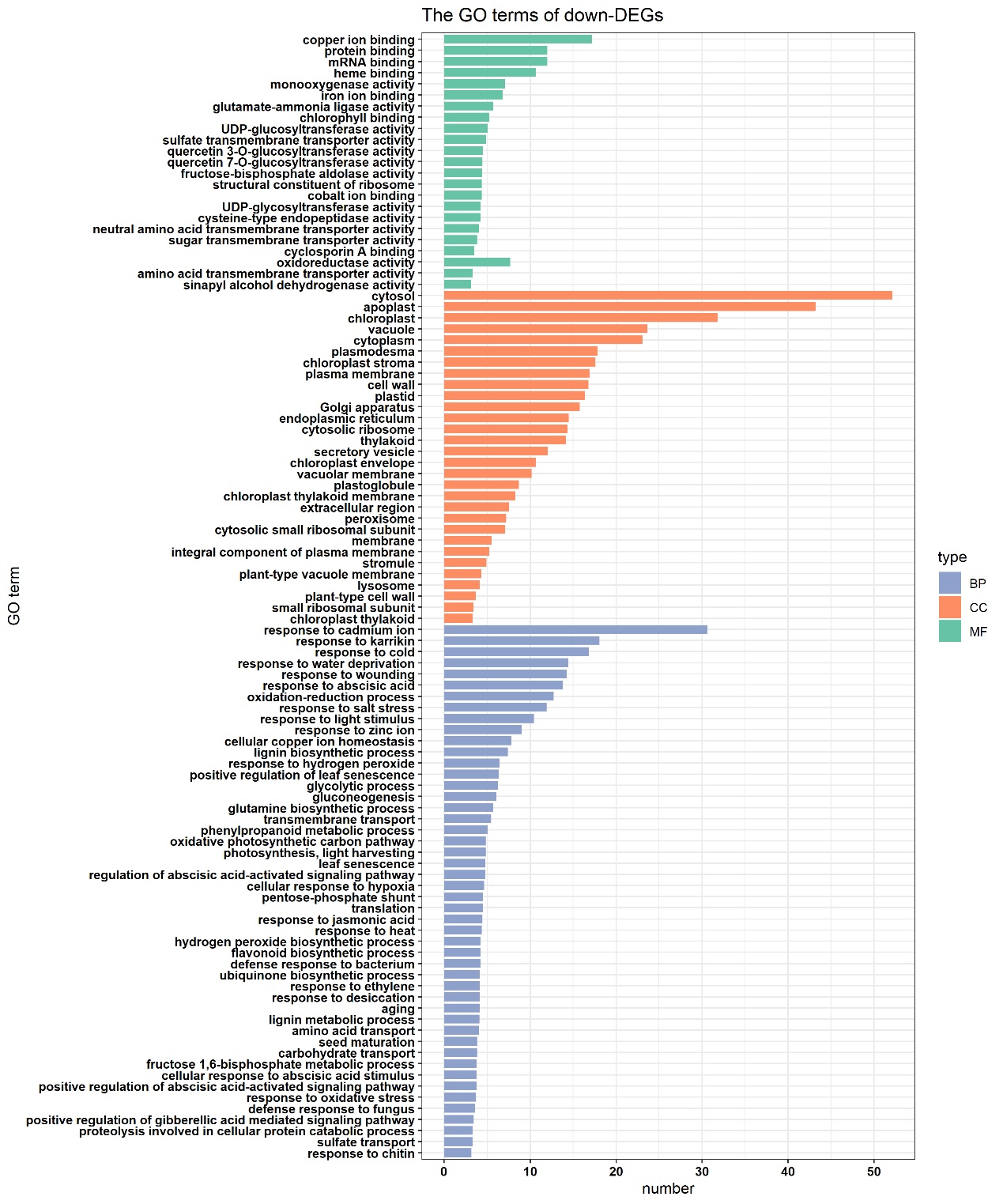


**Fig. S9** GO annotation of all up-and down-regulated genes. (A) GO Annotation Map of up-regulated Genes (B) GO Annotation Map of down-regulated Genes.

A

B

**Fig. S10** Top 20 GO annotation of all up-and down-regulated genes. (A) Top 20 GO Annotation Map of up-DEGs; (B) Top 20 GO Annotation Map of down-DEGs.

A


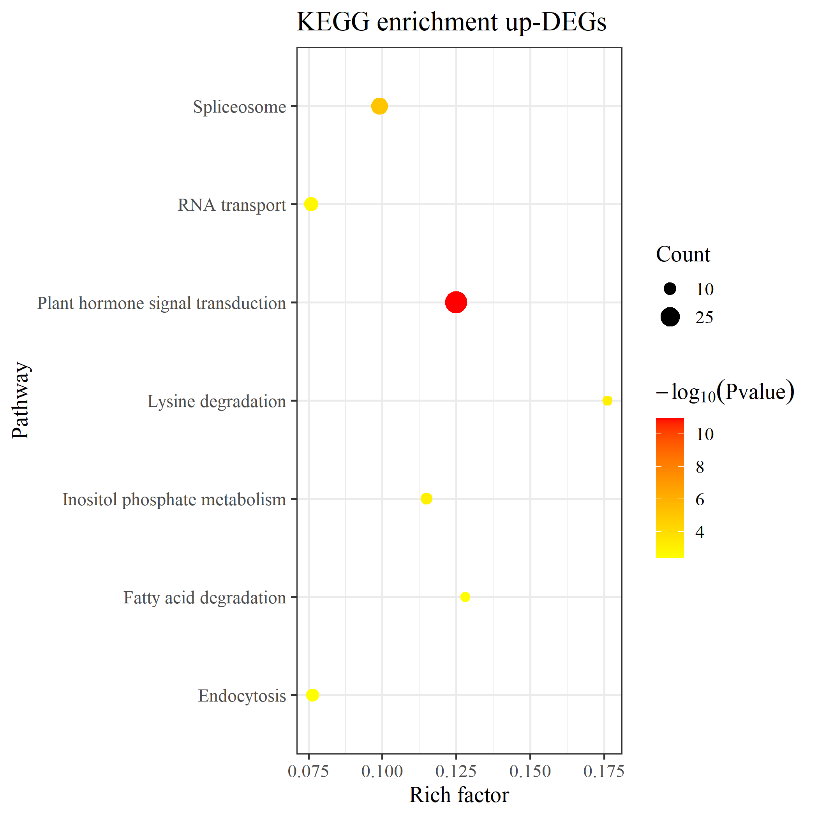


B


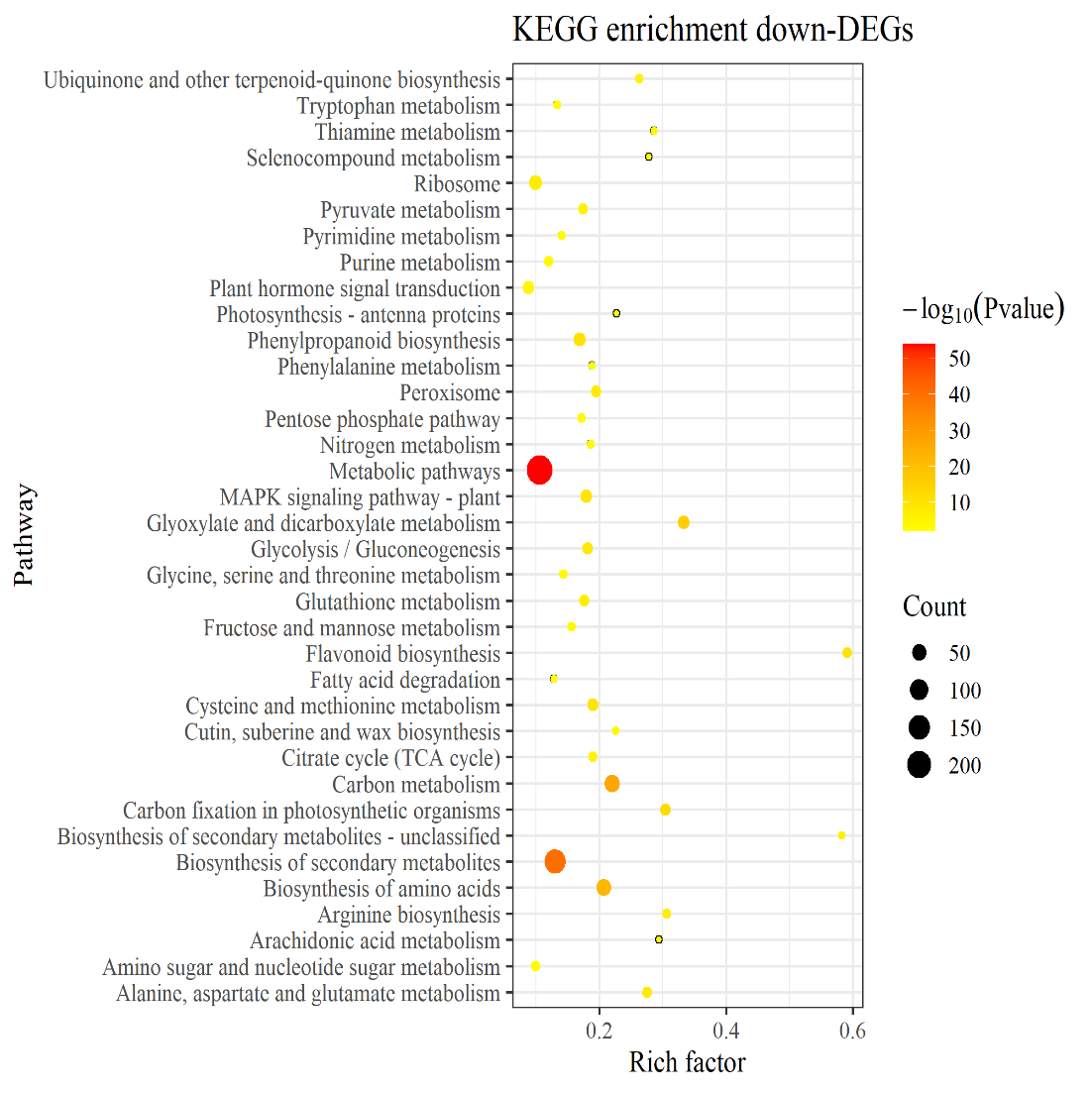


**Fig. S11** KEGG pathway of all up-and down-regulated genes. (A) KEGG pathway of up-DEGs; (B) KEGG pathway of down-DEGs.

A

B

**Fig. S12** **(**A): Correlation of gene expression changes from qRT-PCR and RNA-seq methods of petal. The log2 value of the expression ratio (*aacc*/WT; y-axis) in the 1D analysis was plotted against the log2 fold change from the RNA-seq data (x-axis). The Internal reference of qRT-PCR is *BnaUBC9*. (B): Flavonoid biosynthesis gene expression in petal of WT and the BnaCRTISO double mutant (*aacc*) was determined by qRT-PCR with normalization to *BnaUBC9*.


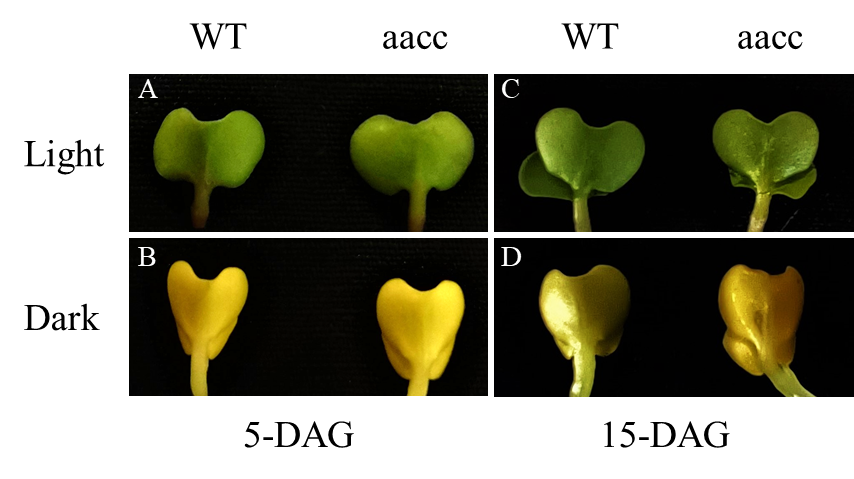


**Fig. S13** The color change of *aacc* cotyledon under light or dark treatment. (A, B): The cotyledon color of *aacc* did not change significantly 5 days after germination (5-DAG) under the light or dark treatment, respectively. (C): The cotyledon color of *aacc* did not change significantly 15 days after germination (15-DAG) under the light treatment. (D): The cotyledon color of *aacc* became orange to the naked eye at 15 days after germination under the dark treatment.
